# Branching in molecular structure enhancement of solubility in CO_2_

**DOI:** 10.1093/pnasnexus/pgad393

**Published:** 2023-11-14

**Authors:** Kazuya Kobayashi, Abbas Firoozabadi

**Affiliations:** Technical Division, INPEX Corporation, Minato-ku, Tokyo 107-6332, Japan; Department of Chemical and Biomolecular Engineering, Rice University, Houston, TX 77005, USA; Department of Chemical and Biomolecular Engineering, Rice University, Houston, TX 77005, USA

**Keywords:** solubility enhancement, CO_2_-philicity, molecular simulations, coarse-grained modeling

## Abstract

Most compounds of some 1,000 amu molecular weight (MW) and higher are poorly soluble in carbon dioxide (CO_2_). Only at very high pressure, there may be mild solubility. This limits the use of CO_2_ as a solvent and modifications of CO_2_ properties through additives. We have developed a coarse-grained molecular model to investigate the dependency of the solubility of hydrocarbon oligomers (MW of ∼1,000 amu) in CO_2_ and on the molecular structure. The coarse-grained model is optimized by the particle swarm optimization algorithm to reproduce density, surface tension, and enthalpy of vaporization of a highly branched hydrocarbon oligomer (poly-1-decene with six repeating units). We demonstrate that branching in molecular structure of oligomers significantly increases solubility in CO_2_. The branching in molecular structure results in up to 270-time enhancement of solubility in CO_2_ than an *n*-alkane with the same MW. The number of structural edges (methyl group) is a key in improved CO_2_-philicity. The solubility of poly-1-decene with nine repeating units (MW of 1,264.4 amu) is higher in CO_2_ than poly-1-dodecene with six repeating units (MW of 1,011.93 amu) because it has more structural edges (10 vs. 7). These results shed light on the enhancement of CO_2_-philicity by altering molecular structure rather than modifying chemical composition in compounds.

**Significance Statement**

There is a considerable interest in highly soluble substances for carbon dioxide (CO_2_), so-called CO_2_-philes. Despite extensive efforts for decades, there is a lack of experiments even for alkanes with a chain length of C_30_ and beyond due to complexities in performing high-pressure experiments. We have developed a coarse-grained molecular model for predicting the solubility of alkanes and oligomers of 1-decene and 1-dodecene with high molecular weight (∼1,000 amu). We demonstrate that higher solubility in CO_2_ can be attained when compounds have a branching structure. This study paves the way toward improving CO_2_-philicity not only by modifying the chemical species but also by altering the molecular structure.

## Introduction

The solubility of compounds in carbon dioxide (CO_2_) is of significant importance in various scientific and industrial fields (1–3). Enhancing the solubility of compounds enables the utilization of CO_2_ as an environmentally friendly solvent (1, 2) and facilitates the modification of CO_2_ properties through additives (3–5). However, the majority of compounds exhibit poor solubility in liquid or supercritical CO_2_, necessitating high pressures even at low concentration (3, 6). Solubility enhancement can be achieved by introducing Lewis base groups or fluoro carbons, altering the chemical composition (2, 5–9). The modification of the chemical composition in the published literature leads to serious environmental and economic burdens, which limits the practical applicability of these compounds (9).

A potential approach to enhance solubility in CO_2_ is through changes in molecular structure. Silva and Orr (10) presented experiments on partitioning in crude oil–CO_2_ systems. Their findings demonstrate that compounds with a branched structure exhibit higher partitioning in CO_2_ compared with compounds with a straight structure for the same molecular weight (MW) (10). The effect becomes less pronounced for carbon chain lengths below 12 (10). Alkanes with a branched structure have higher solubility in CO_2_ than normal alkanes with similar MWs. Various authors have measured the solubility of normal alkanes and branched alkanes (such as squalane) in CO_2_ (11–17). Neopentane (fully branched pentane with four methyl groups) shows about two times higher solubility than *n*-pentane and isopentane; *n*-pentane and isopentane have similar *P*–*x* diagram (14–17) (Fig. S1). Squalane has more than five times higher solubility than normal alkanes with similar MWs (3, 11–13). We have recently conducted molecular simulations to investigate this effect for alkanes up to a carbon number of 20 (18). It is demonstrated that branched hexadecane and eicosane have approximately two to three times higher solubility in CO_2_ than normal hexadecane and eicosane (18). The solubility of decanes is not much affected by molecular structure (18). Methyl-branched surfactants are shown to have greater CO_2_-philicity in hydrocarbon surfactants (5, 19). Theoretical and experimental evidence suggests that the enhancement of solubility in CO_2_ through changes in molecular structure becomes more pronounced when the MW of compounds becomes large. Our current knowledge is limited to carbon chain lengths up to 30. Conducting experimental investigations for higher MWs necessitates high-pressure experiments. Theoretical investigations become challenging due to increased computational costs. The theoretical simulations can play a crucial role in elucidating the structural dependency of solubility in CO_2_ for MWs higher than C_30_.

CO_2_ direct thickeners are compounds that dissolve in CO_2_ to increase its viscosity, thereby enhancing the efficiency of geological sequestration (3–6, 20–28). Practical utilization of CO_2_ direct thickeners is hindered by limited knowledge regarding the solubility in CO_2_. Al-Hinai *et al*. (6) conducted a study demonstrating that only 4 of 27 polymers can dissolve in CO_2_. Among these four polymers, poly-1-decene and poly vinyl ethyl ether are found to effectively thicken CO_2_ (6). The solubility improvement observed in branched alkanes is suggested as a mechanism for the higher solubility of poly-1-decene oligomer, owing to its highly branched structure. To advance the development of CO_2_ direct thickeners, it is crucial to investigate the structural dependency of solubility in CO_2_, particularly for higher MW compounds, including oligomers and beyond.

**Fig. 1.**
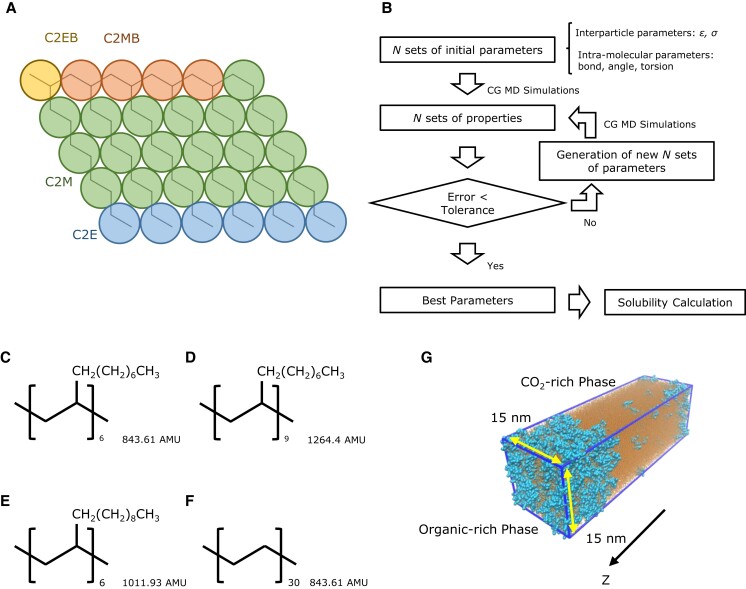
Parameterization of coarse-grained molecular model of poly-1-decene for solubility calculations. A) Mapping scheme and particle type of coarse-grained poly-1-decene with six repeating units (2:1 mapping). B) Flowchart of parameter fitting using PSO method. C) Molecular structure and MW of poly-1-decene with six repeating units (P1D) for solubility calculation. D) Molecular structure and MW of poly-1-decene with nine repeating units (P1D9) for solubility calculation. E) Molecular structure and MW of poly-1-dodecene with six repeating units (P1DD) for solubility calculation. F) Molecular structure and MW of *n*-hexacontane (*n*C_60_) for solubility calculation. G) Interfacial system in solubility calculation. Blue particles and orange particles are oligomers and CO_2_, respectively.

The goal of this work is investigation of the structural dependency of hydrocarbon oligomer solubility in CO_2_. To accomplish this, we have developed a coarse-grained molecular model that can accurately represent branched structures (Fig. 1A). This model is an extension of the transferable coarse-grained model used for normal alkanes (29). The particle swarm optimization (PSO) method is employed to determine interparticle and intramolecular interactions in the model (Fig. 1B). To predict the solubility of various poly-1-decene-type oligomers and examine the influence of molecular structure on CO_2_ solubility (Fig. 1C–F), molecular dynamic simulations are performed using interfacial systems (Fig. 1G).

## Results and discussion

Density, surface tension, and enthalpy of vaporization of poly-1-decene with six repeating units are calculated by all-atom molecular dynamic simulations. Five independent calculations are conducted to obtain better statistics. These are used as reference values during the PSO method. Averages and SEs over the five calculations are summarized in Table 1. The largest SE is 2.9%, which is for surface tension. Time evolution of the systems from the five independent calculations is shown in Fig. S2. Poly-1-decene with six repeating units shows relatively slow relaxation requiring 20 to 30 ns. The last 20 ns of trajectory is used for determining values from each calculation.

**Table 1.** Properties of poly-1-decene with six repeating units from the all-atom model (CGenFF) (30–32) and the coarse-grained model in this study.

Properties	All-atom model	Coarse-grained model	Diff. (%)
Density (kg/m^3^)	838.4 ± 0.3	838.44 ± 0.02	0.01
Surface tension (mN/m)	31.5 ± 0.9	30.8 ± 0.3	2
Enthalpy of vaporization (kJ/mol)	248 ± 3	248.7 ± 0.4	0.2

Error represents SEs.

The coarse-grained molecular parameters representing poly-1-decene oligomers are determined by the PSO method using the reference values from the all-atom molecular dynamic simulations. Parameters for poly-1-decene oligomers are listed in Tables S1–S4. The model is evaluated by five independent calculations using the same system size and the same simulation time as the all-atom molecular dynamic calculations. The procedure ensures that the parameters reproduce properties of the poly-1-decene with six repeating units. The calculations during the PSO method have a smaller number of molecules, shorter simulation time, and only single calculation, which may result in large statistical error. Table 1 shows average and SE over the five calculations for the coarse-grained model. The difference between the all-atom model and the coarse-grained model is at maximum 2%. This difference is less than the SE of the all-atom calculations. We also provide bond length, bond angle, and dihedral angle distributions in Figs. S3–S5, respectively, which show fair agreement with the all-atom model. Transferability of the coarse-grained parameters is examined by conducting all-atom and coarse-grained molecular dynamic simulations for other compounds (poly-1-decene with nine repeating units and poly-1-dodecene with six repeating units; Table S5). The simulations are conducted with the same procedure as the cases of the poly-1-decene with six repeating units, but 70 ns is required for some systems to reach an equilibrium (Figs. S6 and S7). The results show good transferability of our model.

**Fig. 2.**
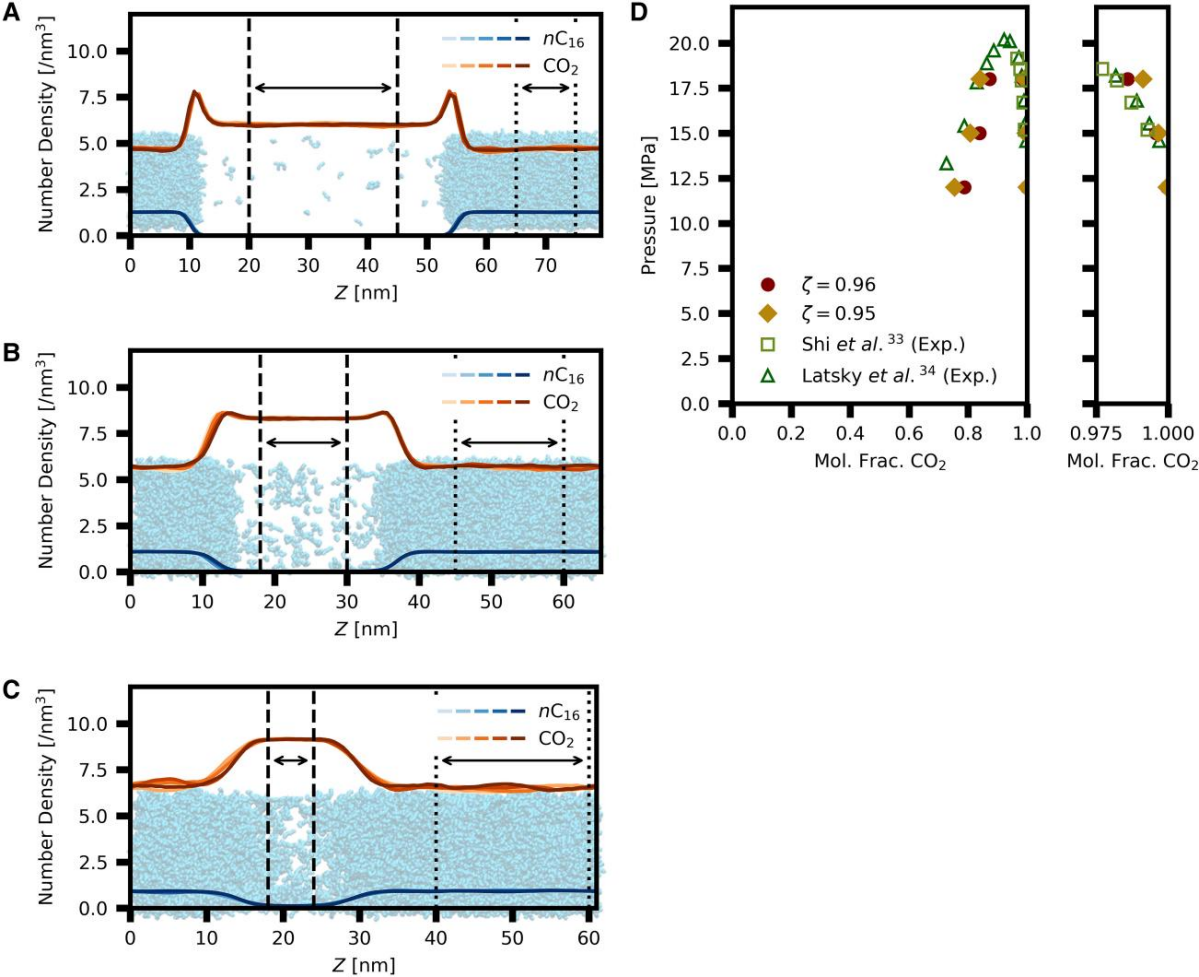
Solubility of *n*-hexadecane. Number density profiles normal to the interface for *n-*hexadecane (*n*C_16_) and CO_2_: mixing parameter zeta (*ζ*) of 0.96, at 344.3 K and at pressures of A) 12 MPa, B) 15 MPa, and C) 18 MPa. Color intensity represents time evolution for every 50 ns blocks, namely 50 ns × 5 blocks. Areas between dashed lines and dotted lines define CO_2_-rich phase and *n*-hexadecane-rich phase. D) *P*−*x* diagram of *n*-hexadecane and CO_2_ system with different mixing parameters and the experimental data at 344.3 K. The right panel represents magnified image of mole fraction of CO_2_ in CO_2_-rich phase (namely, solubility of *n*-hexadecane in CO_2_). SEs are smaller than the symbols.

Mixing rule parameter (*ζ*) is examined by calculating mutual solubility in *n*-hexadecane and CO_2_ binary systems. We find that *ζ* of 0.96 well represents the solubility of *n*-hexadecane in CO_2_. This parameter may not reproduce solubilities in CO_2_ in other conditions because the parameter is fitted for a combination of the force fields used in this study. The mixing rule parameter of 0.95 well reproduces the phase behavior of binary systems of hydrocarbon and CO_2_ for the other combination of force fields (TraPPE-UA alkanes and TraPPE-small CO_2_) (18). Figure 2A–C shows number density distribution normal to interfaces at pressures of 12, 15, and 18 MPa, respectively, for the mixing rule parameter of 0.96. The figures represent time evolution 5 × 50 ns blocks by color intensities. Dashed lines and dotted lines define CO_2_-rich phase and *n*-hexadecane-rich phase to calculate mutual solubility. For the bulk phase, we include the area where density is constant to exclude the interfacial region. The number density distributions in Fig. S8 are based on the mixing rule parameter of 0.95. Low system size dependency is confirmed by conducting simulation at 18 MPa, which has double the number of molecules, while interfacial area is fixed (Fig. S9). In addition to increase in computational cost due to larger number of molecules, the extension of system size normal to the interface results in longer relaxation time (three times for our case) because the components diffuse longer distances in the system. Figure 2D presents *P*–*x* diagram for simulations results and experimental data (33, 34). The mole fractions of CO_2_ in CO_2_-rich phase agree with experimental data for *ζ* of 0.96 especially at high pressure (18 MPa; circles in the magnified panel in Fig. 2D), whereas the mole fractions of CO_2_ in *n*-hexadecane-rich phase is represented better by *ζ* of 0.95 than by *ζ* of 0.96 (circles and diamonds in Fig. 2D). A *ζ* of 0.96 better describes the CO_2_-rich phase and therefore solubility of oligomers in CO_2_.

**Fig. 3.**
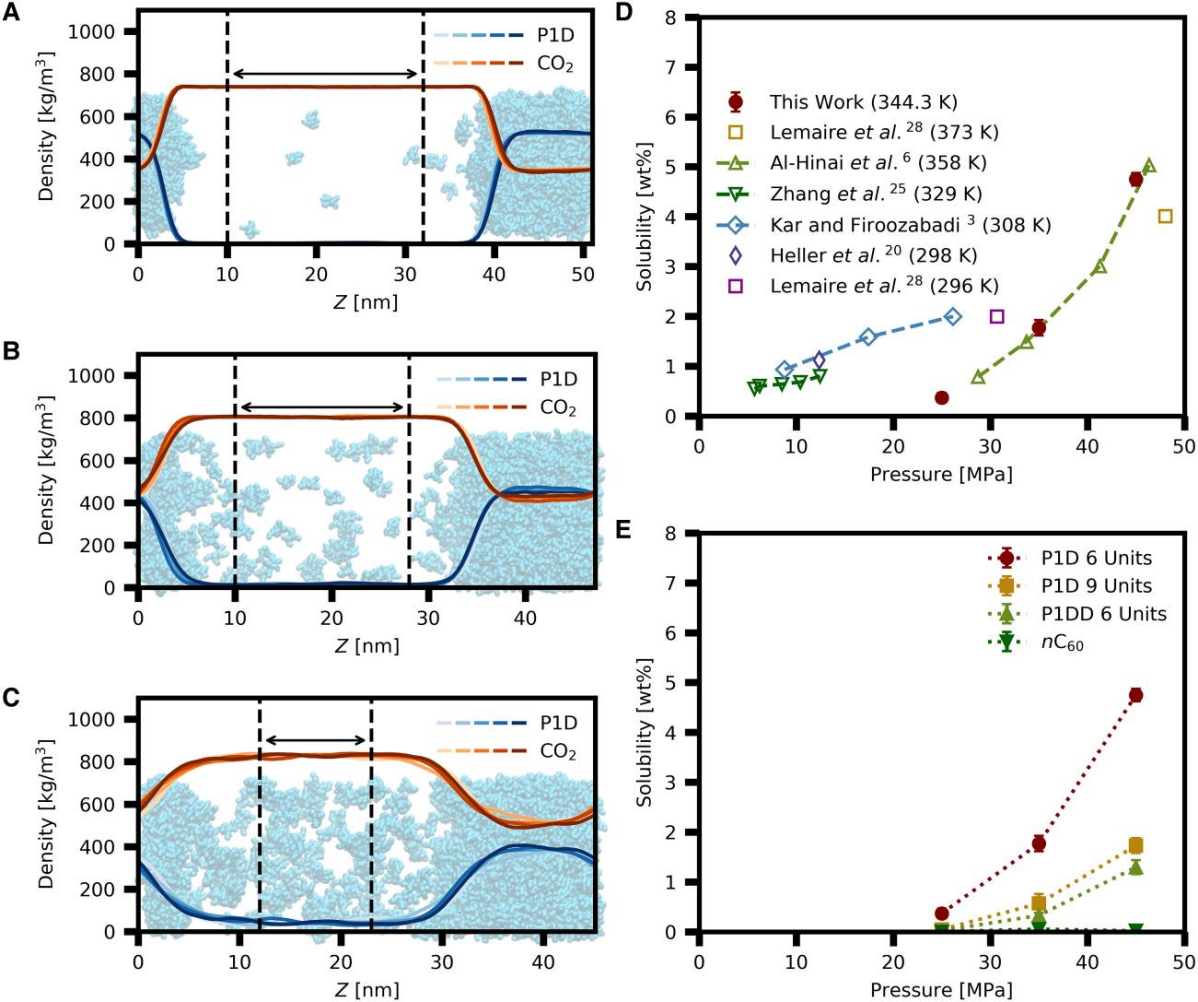
Solubility of poly-1-decene oligomers, a poly-1-dodecene oligomer, and *n*-hexacontane. Density profiles normal to the interface for the system of poly-1-decene with six repeating units and CO_2_ at 344.3 K and at pressures of (A) 25 MPa, (B) 35 MPa, and (C) 45 MPa. Color intensity represents time evolution for every 100 ns blocks, namely 100 ns × 5 blocks. Areas between dashed lines define CO_2_-rich phase from which solubility is calculated. Snapshots from the last configuration are overlaid, where CO_2_ particles are not shown for clarity. D) Solubility of poly-1-decene with six repeating units and experimental data. E) Structural dependency of solubility of oligomers at 344.3 K. Error bars represent SEs.

We use interfacial systems to model the solubility of poly-1-decene (Fig. 1G). Figure 3A–C presents density distributions normal to interface for poly-1-decene with six repeating units and CO_2_ at pressures of 25, 35, and 45 MPa, respectively. The CO_2_-rich phase and poly-1-decene–rich phase are both modeled. Solubility for this system is overlaid to experimental data (Fig. 3D). One consideration in molecular simulations of oligomers is slow relaxation time. We investigate initial configuration dependency for poly-1-decene with six repeating units at a pressure of 35 MPa (Fig. S10). Our calculations indicate fully relaxed state by simulation time of 1.1 µs. Initial configuration with uniform density distribution of poly-1-decene with six repeating units and CO_2_, namely super-saturated solution, yields the same solubility as the separate pure–pure initial configuration. Our model reproduces the solubility of poly-1-decene oligomers in CO_2_. The results are within the variation of the experimental results, especially align with Al-Hinai *et al*. (6) (up-pointing triangles in Fig. 3D). We may underestimate the solubility at a pressure of 25 MPa. It must be emphasized that compounds used in the experiments may have mixed polymerization degrees (i.e. average MW corresponds to MW of poly-1-decene with six repeating units). Dissipative particle dynamic simulations with poly-1-decene with six repeating units also show the same trend as ours, namely sharp increase in solubility as increasing pressure (35). Different authors may not have used the same chemical in their experiments. We are conducting nuclear magnetic resonance (NMR) analysis to find out the distribution of repeating units of the oligomers used in the experiments. The effect on solubility in CO_2_ with multipolymerization degree will be investigated in future.

Figure 3E indicates the structural dependency of solubility in CO_2_ for poly-1-decene type of oligomers. Density profiles normal to interface other than poly-1-decene with six repeating units are provided in Figs. S11–S13. We compare the solubilities of poly-1-decene with six repeating units (circles in Fig. 3E and Fig. 1C for molecular structure) and *n*-hexacontane (down-pointing triangles in Fig. 3E and Fig. 1F for molecular structure) in CO_2_. The two compounds have the same MW. Branching in structure significantly increases solubility in CO_2_. The solubility increases by 35 and 267 times at pressures of 35 and 45 MPa, respectively. *n*-Hexacontane is waxy solid at 344.3 K, while poly-1-decene with six repeating units is liquid at this temperature. We have conducted the solubility calculations of *n-*hexacontane at 385.0 K, which is above the melting point (Fig. S14). The results are close at the two temperatures.

As pointed out earlier, improved solubility by branching structure is observed experimentally and theoretically up to a carbon number of 30 (18). This effect becomes significant as MW increases. We have demonstrated that the solubility increases two to three times in eicosanes (MW of 282.5 amu), *n*-eicosane and 2,2,4,6,6,8,10,10-nonmethylundecane (a structural isomer of *n*-eicosane) (18). In this work, we establish that solubility improvement by branching becomes significant when MW is large. Change in molecular structure, namely branching, rather than change in chemical composition can be an alternative strategy for the development of CO_2_-soluble oligomer/polymer. The advantage here for the application of direct thickeners for CO_2_ geological storage is that branching does not lead to adsorption but change in chemical composition may promote adsorption to minerals (4).

Next, the effects of increasing repeating units and increasing length of branching part (branching length) on solubility are investigated (squares and up-pointing triangles in Fig. 3E). Both decrease solubility in CO_2_ mainly due to increase in MW in comparison with poly-1-decene with six repeating units (circles in Fig. 3E). The solubility becomes lower with an increase in branching length than adding units. The solubility of poly-1-decene with nine units (squares in Fig. 3E and Fig. 1D for molecular structure) is higher than that of poly-1-dodecene with six repeating units (up-pointing triangles in Fig. 3E and Fig. 1E for molecular structure). The MW of poly-1-decene with nine repeating units (1,264.4 amu) is higher than that of poly-1-dodecene with six repeating units (1,011.93 amu). We point out that number of structural edges (namely methyl group) affect solubility in CO_2_. Molecular dynamic simulations demonstrate that a methyl group can be surrounded by a larger number of CO_2_ molecules than methylene group (3, 18). The difference in solvation degree is a mechanism of solubility enhancement. We analyze the solvation structure of poly-1-decene with six repeating units by radial distribution function and coordination numbers (Fig. S15). The structural edges (C2E) have greater affinity to CO_2_ than that of middle of chains (C2M); this results in larger coordination number for the structural edges. Our simulations are in line with experimental observations that methyl-branched surfactants have greater CO_2_-philicity (5, 19). Increase in branching length results in higher MW, while number of edge group is kept the same. Adding repeating units results in increase in both MW and number of edge group. This explains the difference in the effect on solubility by branching length and number of repeating units.

We have developed coarse-grained model of poly-1-decene oligomers to investigate solubility in CO_2_. The model reproduces density, surface tension, and enthalpy of vaporization calculated by all-atom model. Theoretical prediction of the solubility of poly-1-decene with six repeating units agrees with experimental data. The coarse-grained model can be used to predict structural dependency of solubility in CO_2_ extending to oligomer to polymer scale. Structural dependency of solubility in CO_2_ is investigated for oligomers. It is demonstrated that branching significantly increases solubility in CO_2_; a compound with branched structure (oligomer of poly-1-decene) shows 35 and 267 times at 35 and 45 MPa, respectively, larger solubility than that of straight chain structure with the same MW. This study sets the stage in attaining CO_2_-philicity through branched structured without altering chemical compositions.

## Materials and methods

### Molecular dynamic simulations

GROMACS (36) is used in molecular dynamic simulations in this study. Temperature and pressure are controlled stochastically. For bulk systems, a cutoff length of 1.4 nm is applied for nonbonded interactions. In solubility calculations of binary interfacial systems, a cutoff length of 2.4 nm is found necessary because we find bulk density changes with shorter cutoff lengths. Analytical tail correction is applied for treating long-range dispersion potential. A previous study has highlighted the need for longer cutoff lengths in certain anisotropic systems (37). Particle mesh Ewald summation (38) is utilized to calculate long-range electrostatic interactions with short-range cutoff of 1.4 nm. In this study, the following mixing rule is implemented:


(1) $$\epsilon_{ij}=\zeta \sqrt{\epsilon_{ii}\epsilon_{jj}}$$

(2) $$\sigma_{ij}=\frac{\sigma_{ii}+\sigma_{jj}}{2}$$

The value of *ζ* is set to 1 for unlike particles of the same type of molecule, ensuring the preservation of pure system properties. Mutual solubility may exhibit significant dependency on the value of *ζ*. In this study, we examine the dependency of mutual solubility on this parameter using binary systems of *n*-hexadecane and CO_2_.

CGenFF (30–32) is selected as an all-atom force field for modeling poly-1-decene with six repeating units. This force field is shown to accurately reproduce the properties of hydrocarbons (39). To obtain reference properties for fitting purposes, all-atom molecular dynamic simulations are conducted. These simulations include the density, surface tension, and enthalpy of vaporization. The enthalpy of vaporization encompasses the potential energy of both the liquid and vapor phases. The theoretical background for calculating the surface tension and enthalpy of vaporization is provided in the SI Appendix. The simulations are performed at a temperature of 300 K and a pressure of 0.1 MPa, following the conditions previously established for coarse-grained normal alkanes (29). The systems consist of 500 molecules for the density and surface tension calculations, as well as for determining the potential energy of the liquid phase, which is utilized for the calculation of the enthalpy of vaporization. Additionally, a single-molecule system is used to calculate the potential energy of the vapor phase. Each simulation has a duration of 50 ns, employing a timestep of 1 fs.

Interfacial systems are employed to predict solubility in this study (Fig. 1G). For oligomers, combination of the model developed by An *et al*. (29) and the newly developed coarse-grained model are utilized. Force field for CO_2_ is a single-site model proposed by Higashi *et al*. (40). The model reproduces pressure dependency of density of CO_2_ (41) and is used to investigate the phase behavior of binary systems (40, 42). To prepare initial configurations, pure CO_2_ and pure oligomer systems are individually equilibrated under the conditions required for solubility calculations. A temperature of 344.3 K is used in these simulations. The binary systems of *n*-hexadecane and CO_2_ consist of 10,000 *n*-hexadecane molecules and 100,000 CO_2_ molecules. The mixing rule (Eqs. 1 and 2) is examined using this system. For the solubility calculations of oligomers and *n*-hexacontane, the systems are composed of 1,200 oligomers/*n*-hexacontane and 100,000 CO_2_ molecules. Simulations are carried out for a duration of 0.6 µs for the *n*-hexadecane systems and 1.1 µs for the oligomer systems using a timestep of 4 fs. The last 250 and 500 ns of trajectories are analyzed for the *n*-hexadecane and the oligomer systems, respectively. SEs are defined by dividing the last trajectories into five blocks (e.g. 100 ns × 5 blocks for the oligomer systems). Visual molecular dynamics is used to present the snapshots of systems (43).

### Coarse-grained model

The nonbonded interactions in the coarse-grained model are described by the Lennard–Jones potential:


(3) $$U_{ij}^{\mathrm{LJ}}=4\epsilon_{ij}\left({\left(\frac{\sigma_{ij}}{r_{ij}}\right)}^{12}-{\left(\frac{\sigma_{ij}}{r_{ij}}\right)}^{6}\right)$$

where $\epsilon_{ij}$ and $\sigma_{ij}$ are parameters of the Lennard–Jones potential, and $r_{ij}$ represents the distance between particles *i* and *j*. Electrostatic interactions are not considered in this particular model. The bond, angle ( $\theta_{ijk}$), and dihedral angle (*ϕ*) interactions are represented as follows:


(4) $$U^{\mathrm{bond}}=\frac{1}{2}k_{ij}^{b}(r_{ij}-b_{ij}{)}^{2}$$

(5) $$U^{\mathrm{angle}}=\frac{1}{2}k_{ijk}^{\theta }(\theta_{ijk}-\theta_{ijk}^{0}{)}^{2}$$

(6) $$U^{\mathrm{dihedral}}=\underset{n}{\sum }\;k_{n}^{\phi }(1+\cos\;(n\phi -\phi_{n}^{s}))$$

$k_{ij}^{b}$ and $b_{ij}$ represent the bond force constant and the equilibrium bond length, respectively. $k_{ijk}^{\theta }$ is the angle force constant, and $\theta_{ijk}^{0}$ is the equilibrium angle. $k_{n}^{\phi }$ is the dihedral force constant. In a previous study on coarse-grained modeling for normal alkanes, it has been assumed that dihedral angle interactions are not necessary (29). In this study, we find that the dihedral angle distributions are uniform when four particles have two center particles, where the particle type is derived from normal alkanes, specifically *X*-C2M-C2M-*X* (where *X* represents any particle type; Fig. S16A). This confirmation supports the assumption made in a previous study (29). However, we have observed nonuniformity in the dihedral angle distributions for the other cases (Fig. S16B). This highlights the critical role of dihedral angle interactions in accurately representing the molecular shape of poly-1-decene.

### Particle swarm optimization

The PSO method is employed to determine the parameters of the coarse-grained poly-1-decene model (Fig. 1B and SI Appendix). A swarm size of 200 is selected, resulting in a total of 600 coarse-grained molecular dynamic simulations per iteration considering the pure liquid system, interfacial system, and vapor system. The coarse-grained molecular dynamic simulations are conducted with a duration of 5 ns, using a timestep of 5 fs, and a system size of 50 molecules of poly-1-decene with six repeating units. Initial configurations are prepared by mapping the coordinate of corresponding center of mass from final configurations of the all-atom molecular dynamic simulations. Pressure and temperature conditions are kept consistent with the all-atom molecular dynamic simulations. Initial parameters for the bond length, bond angle, and the dihedral angle interactions are determined based on the Boltzmann assumption using the results obtained from the all-atom molecular dynamic simulations. Subsequently, these parameters are allowed to change by ±10% during the PSO iterations. For the initial guess of the Lennard–Jones parameters, random values within the predefined boundaries are chosen. The specific details of the boundary and the initial guess are presented in Table S6. Finally, the optimized parameters are evaluated by performing calculations with a duration of 50 ns, a timestep of 5 fs, and a system size of 500 molecules. This is done to eliminate a potential system size or simulation time dependency during the PSO iterations.

## Supplementary Material

pgad393_Supplementary_Data

## Data Availability

The authors confirm that the data supporting the findings of this study are available within the article and its Supplementary material online.

## References

[1] Sarbu T , Styranec T, Beckman EJ. 2000. Non-fluorous polymers with very high solubility in supercritical CO_2_ down to low pressures. Nature. 405:165–168. 10821268 10.1038/35012040

[2] Girard E , Tassaing T, Marty J-D, Destarac M. 2016. Structure-property relationships in CO_2_-philic (co)polymers: phase behavior, self-assembly, and stabilization of water/CO_2_ emulsions. Chem Rev. 116:4125–4169. 27014998 10.1021/acs.chemrev.5b00420

[3] Kar T , Firoozabadi A. 2022. A effective viscosification of supercritical carbon dioxide by oligomers of 1-decene. iScience. 25:104266. 35521540 10.1016/j.isci.2022.104266 PMC9062731

[4] Afra S , Alhosani M, Firoozabadi A. 2023. Improvement in CO_2_ geo-sequestration in saline aquifers by viscosification: from molecular scale to core scale. Int J Greenh Gas Control. 125:103888.

[5] Cummings S , Trickett K, Enick R, Eastoe J. 2011. CO_2_: a wild solvent, tamed. Phys Chem Chem Phys. 13:1276–1289. 20648383 10.1039/c003856c

[6] Al-Hinai NM , et al 2018. Experimental evaluations of polymeric solubility and thickeners for supercritical CO_2_ at high temperatures for enhanced oil recovery. Energy Fuels. 32:1600–1611.

[7] Rindfleisch F , DiNoia TP, Mchugh MA. 1996. A solubility of polymers and copolymers in supercritical CO_2_. J Phys Chem. 100:15581–15587.

[8] Girard E , et al 2012. Enhancement of poly(vinyl ester) solubility in supercritical CO_2_ by partial fluorination: the key role of polymer-polymer interactions. J Am Chem Soc. 134:11920–11923. 22780868 10.1021/ja304585d

[9] Sarbu T , Styranec TJ, Beckman EJ. 2000. Design and synthesis of low cost, sustainable CO_2_-philes. Ind Eng Chem Res. 39:4678–4683.

[10] Silva MK , Orr FM Jr. 1987. Effect of oil composition on minimum miscibility pressure-part 1: solubility of hydrocarbons in dense CO_2_. SPE Res Eng. 2:468–478.

[11] Chandler K , Pouillot FLL, Eckert CA. 1996. Phase equilibria of alkanes in natural gas systems. 3. Alkanes in carbon dioxide. J Chem Eng Data. 41:6–10.

[12] Sovová H , Jez J, Khachaturyan M. 1997. Solubility of squalane, dinonyl phthalate and glycerol in supercritical CO_2_. Fluid Phase Equilib. 137:185–191.

[13] Reverchon E , Russo P, Stassi A. 1993. Solubilities of solid octacosane and triacontane in supercritical carbon dioxide. J Chem Eng Data. 38:458–460.

[14] Besserer GJ , Robinson DB. 1973. Equilibrium-phase properties of *n*-pentane-carbon dioxide system. J Chem Eng Data. 18:416–419.

[15] Cheng H , Fernández MEP, Zollweg JA, Streett WB. 1989. Vapor-liquid equilibrium in the system carbon dioxide + *n*-pentane from 252 to 458 K at pressures to 10 MPa. J Chem Eng Data. 34:319–323.

[16] Besserer GJ , Robinson DB. 1975. Equilibrium-phase properties of isopentane-carbon dioxide system. J Chem Eng Data. 20:93–96.

[17] Shah NN , Fernández MEP, Zollweg JA, Streett WB. 1990. Vapor-liquid equilibrium in the system carbon dioxide + 2,2-dimethylpropane from 262 to 424 K at pressures to 8.4 MPa. J Chem Eng Data. 35:278–283.

[18] Kobayashi K , Firoozabadi A. 2022. Effect of branching on mutual solubility of alkane-CO_2_ systems by molecular simulations. J Phys Chem B. 126:8300–8308. 36197719 10.1021/acs.jpcb.2c05774

[19] Eastoe J , et al 2003. Micellization of economically viable surfactants in CO_2_. J Colloid Interface Sci. 258:367–373. 12618107 10.1016/s0021-9797(02)00104-2

[20] Heller JP , Dandge DK, Card RJ, Donaruma LG. 1985. Direct thickeners for mobility control of CO_2_ floods. Soc Pet Eng J. 25:679–686.

[21] Bae JH , Irani CA. 1993. A laboratory investigation of viscosified CO_2_ process. SPE Adv Technol Ser. 1:166–171.

[22] Huang Z , et al 2000. Enhancement of the viscosity of carbon dioxide using styrene/fluoroacrylate copolymers. Macromolecules. 33:5437–5442.

[23] Xu J , Enick RM. 2003. Thickening carbon dioxide with the fluoroacrylate-styrene copolymer. SPE J. 8:85–91.

[24] Kilic S , Enic RM, Beckman EJ. 2019. Fluoroacrylate-aromatic acrylate copolymers for viscosity enhancement of carbon dioxide. J Supercrit Fluids. 146:38–46.

[25] Zhang S , She Y, Gu Y. 2011. Evaluation of polymers as direct thickeners for CO_2_ enhanced oil recovery. J Chem Eng Data. 56:1069–1079.

[26] Sun W , et al 2018. Thickening supercritical CO_2_ with π-stacked copolymers: molecular insights into the role of intermolecular interaction. Polymers (Basel). 10:268. 30966303 10.3390/polym10030268 PMC6414866

[27] Chen R , et al 2020. Evaluation of CO_2_-phylicity and thickening capability of multichain poly (ether-carbonate) with assistance of molecular simulations. J Appl Polym Sci. 138:49700.

[28] Lemaire PC , Alenzi A, Lee JJ, Beckman EJ, Enick RM. 2021. Thickening CO_2_ with direct thickeners, CO_2_-in oil emulsions, or nanoparticle dispersions: literature review and experimental validation. Energy Fuels. 35:8510–8540.

[29] An Y , Bejagam KK, Deshmukh SA. 2018. Development of new transferable coarse-grained models of hydrocarbons. J Phys Chem B. 122:7143–7153. 29928806 10.1021/acs.jpcb.8b03822

[30] Vanommeslaeghe K , et al 2010. CHARMM general force field: a force field for drug-like molecules compatible with the CHARMM all-atom additive biological force field. J Comput Chem. 31:671–690. 19575467 10.1002/jcc.21367 PMC2888302

[31] Vanommeslaeghe K , MacKerell AD Jr. 2012. Automation of the CHARMM general force field (CGenFF) I: bond perception and atom typing. J Chem Inf Model. 52:3144–3154. 23146088 10.1021/ci300363c PMC3528824

[32] Vanommeslaeghe K , Raman EP, MacKerell AD Jr. 2012. Automation of the CHARMM general force field (CGenFF) II: assignment of bonded parameters and partial atomic charges. J Chem Inf Model. 52:3155–3168. 23145473 10.1021/ci3003649 PMC3528813

[33] Shi Q , Jing L, Qiao W. 2015. Solubility of *n*-alkanes in supercritical CO_2_ at diverse temperature and pressure. J CO_2_ Util. 9:29–38.

[34] Latsky C , Cordeiro B, Schwartz CE. 2020. High pressure bubble- and dew-point data for systems containing CO_2_ with 1-decanol and *n*-hexadecane. Fluid Phase Equilib. 521:112702.

[35] Goicochea AG , Firoozabadi A. 2019. Atomistic and mesoscopic simulations of the structure of CO_2_ with fluorinated and nonfluorinated copolymers. J Phys Chem C. 123:17010–17018.

[36] Bauer P , Hess B, Lindahl E. GROMACS 2022.1. Zenodo [deposited 22 Apr 2022]. 10.5281/zenodo.6451567.

[37] Cao J , et al 2019. Molecular simulation of CH_4_ adsorption behavior in slit nanopores: verification of simulation methods and models. AIChE J. 65:16733.

[38] Darden T , York D, Pedersen L. 1993. Particle mesh Ewald: an *N*log(*N*) method for Ewald sums in large systems. J Chem Phys. 98:10089–10092.

[39] Caleman C , et al 2012. Force field benchmark of organic liquids: density, enthalpy of vaporization, heat capacities, surface tension, isothermal compressibility, volumetric expansion coefficient, and dielectric constant. J Chem Theory Comput. 8:61–74. 22241968 10.1021/ct200731v PMC3254193

[40] Higashi H , Iwai Y, Uchida H, Arai Y. 1998. Diffusion coefficients of aromatic compounds in supercritical carbon dioxide using molecular dynamics simulation. J Supercrit Fluids. 13:93–97.

[41] Senapati S , et al 2002. Structure of phosphate fluorosurfactant based reverse micelles in supercritical carbon dioxide. Langmuir. 18:7371–7376.

[42] Du Q , Yang Z, Yang N, Yang X. 2010. Coarse-grained model for perfluorocarbons and phase equilibrium simulation of perfluorocarbons/CO_2_ mixtures. Ind Eng Chem Res. 49:8271–8278.

[43] Humphrey W , Dalke A, Schulten K. 1996. VMD: visual molecular dynamics. J Mol Graph. 14:33–38. 8744570 10.1016/0263-7855(96)00018-5

