# Supplementary material for: Branching in molecular structure enhancement of solubility in CO2
Source: PNAS Nexus. 2023 Nov 14;2(11):pgad393. doi: 10.1093/pnasnexus/pgad393 (PMC10675989; doi:10.1093/pnasnexus/pgad393)
Supplement: pgad393_Supplementary_Data [file pgad393_supplementary_data.zip › 20231120PNAS_SI_Kobayashi_Firoozabadi_clean.docx]

**Supporting Information for**

Branching in molecular structure enhancement of solubility in CO_2_.

Kazuya Kobayashi, Abbas Firoozabadi

Kazuya Kobayashi, Abbas Firoozabadi

Email: [kazuya.kobayashi@inpex.co.jp](mailto:kazuya.kobayashi@inpex.co.jp), [abbas.firoozabadi@rice.edu](mailto:abbas.firoozabadi@rice.edu)

**This PDF file includes:**

Supplementary Methods

Figures S1 to S16

Tables S1 to S6

Supplementary Methods

**Enthalpy of Vaporization.** Enthalpy of vaporization ($\Delta H_{V}$) is calculated by potential energies in vapor phase and liquid phase:

| $\Delta H_{V}\left( T \right)=U^{\mathrm{vap}}\left( T \right)-U^{\mathrm{liq}}\left( T \right)+RT$ | (S1) |
| --- | --- |

$U^{\mathrm{vap}}$ is potential energy of vapor phase and $U^{\mathrm{liq}}$ is potential energy of liquid phase. $R$ represents the gas constant. This study calculates $U^{\mathrm{vap}}$ and $U^{\mathrm{liq}}$ separately. $U^{\mathrm{vap}}$ is determined by a system consisting of a single molecule while keeping volume of the system constant ($NVT$ ensemble). $U^{\mathrm{liq}}$ is estimated from the system with multiple molecules controlling temperature and pressure ($NPT$ ensemble). This also provides the density of the system.

**Surface Tension.** Surface tension is calculated by $NVT$ ensemble of a slab system. The system is constructed by expanding the $z$-axis of equilibrated cubic system. Surface tension ($\gamma$) of the system is represented by components of pressure tensors ($P_{xx}$, $P_{yy}$, $P_{zz}$):

| $\gamma=\frac{L_{z}}{2}\left\langle P_{zz}-\frac{P_{xx}+P_{yy}}{2} \right\rangle$ | (S2) |
| --- | --- |

$L_{z}$ is the length of a box in the $z$-direction. We use $L_{z}$ of 20 nm, which yields approximately *z*-length of 10 nm for liquid phase and *z*-length of 10 nm for vapor phase, in this study.

**Particle Swarm Optimization (PSO) Algorithm.** The PSO algorithm is based on iterative optimization updating from the influence of global best parameters and personal best parameters. Mathematical form of updating parameters ($\mathbf{x}_{n}$) is shown as following:

| $\mathbf{v}_{n+1}=w\mathbf{v}_{n}+c_{1}\mathbf{r}_{1}\left( \mathbf{x}_{n}^{\mathrm{gbest}}\boldsymbol{-}\mathbf{x}_{n} \right)\boldsymbol{+}c_{2}\mathbf{r}_{2}\left( \mathbf{x}_{n}^{\mathrm{pbest}}\boldsymbol{-}\mathbf{x}_{n} \right)$ | (S3) |
| --- | --- |
| $\mathbf{x}_{n+1}\boldsymbol{=}\mathbf{x}_{n}+\mathbf{v}_{n+1}$ | (S4) |

$n$ represent number of iterations. $\mathbf{r}_{1}$ and $\mathbf{r}_{2}$ are random numbers between 0 and 1. $w$ is inertia factor. $c_{1}$ and $c_{2}$ are constants which determine the factor that the particles are pulled towards swarm global best ($\mathbf{x}_{n}^{\mathrm{gbest}}$) and their personal best ($\mathbf{x}_{n}^{\mathrm{pbest}}$). We use $c_{1}$, $c_{2}$, $w$ of 0.6, 0.3, 0.4, respectively. The error minimized during the PSO algorithm is represented by the following expression.

| $E=\sum_{i} \left( \frac{y_{i}^{\mathrm{CG}}\left( \mathbf{x} \right)-y_{i}^{\mathrm{Ref}}}{y_{i}^{\mathrm{Ref}}} \right)^{2}$ | (S5) |
| --- | --- |


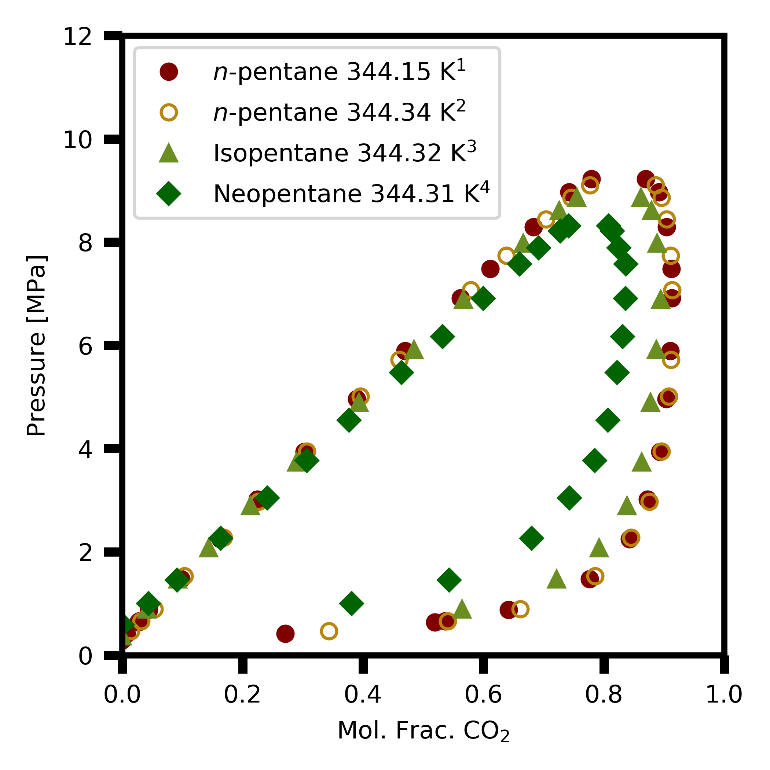


Fig. S1. $\boldsymbol{P}$-$\boldsymbol{x}$ diagrams of *n*-pentanes and its structural isomers (isopentane and neopentane).


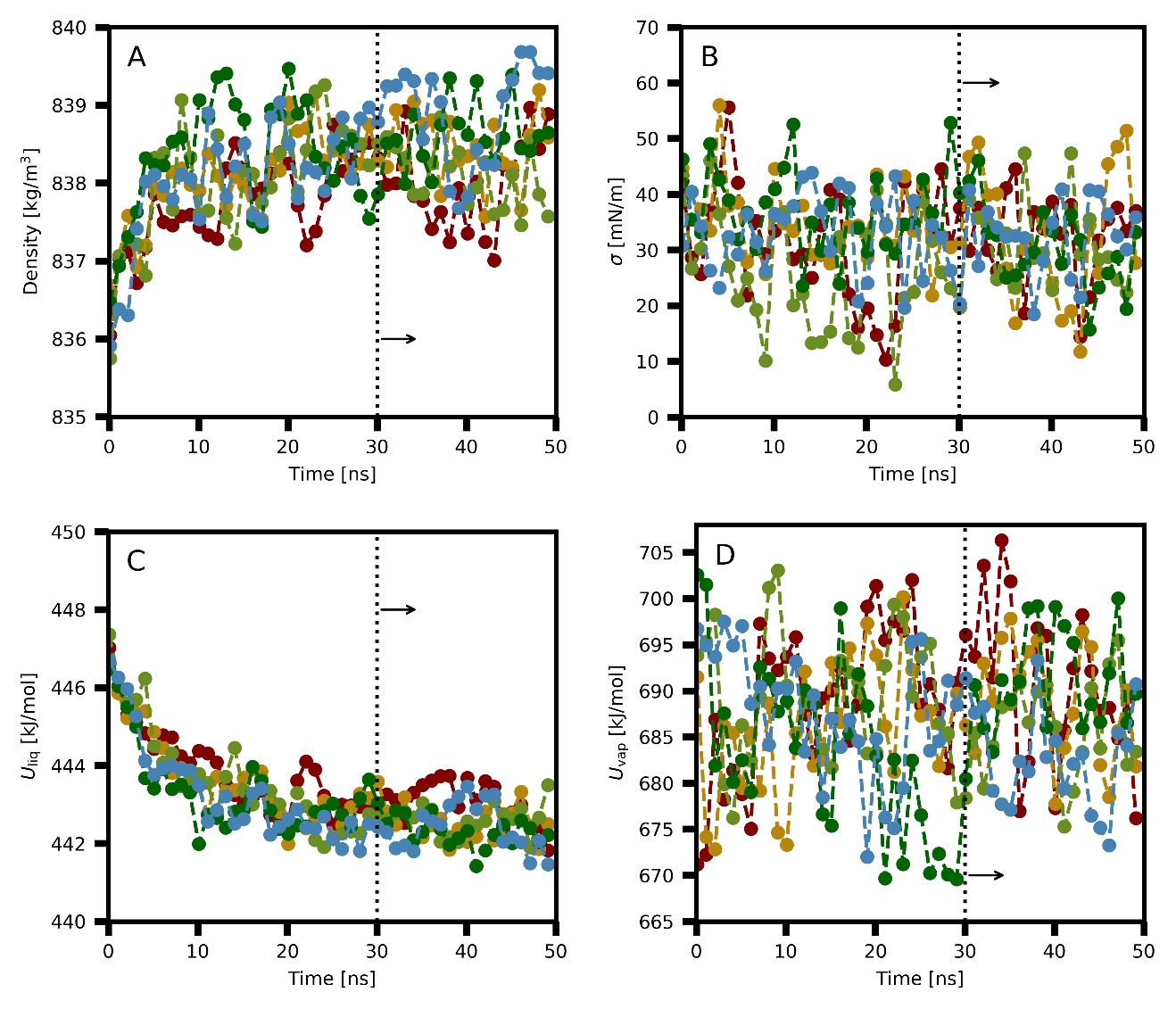


Fig. S2. Time evolutions of (A) density, (B) surface tension, (C) potential energy of liquid phase, and (D) potential energy of vapor phase calculated by five independent all-atom molecular dynamics simulations for poly 1-decene with six repeating units. Reference properties are obtained from the average of the last 20 ns calculations represented by dotted lines and arrows.


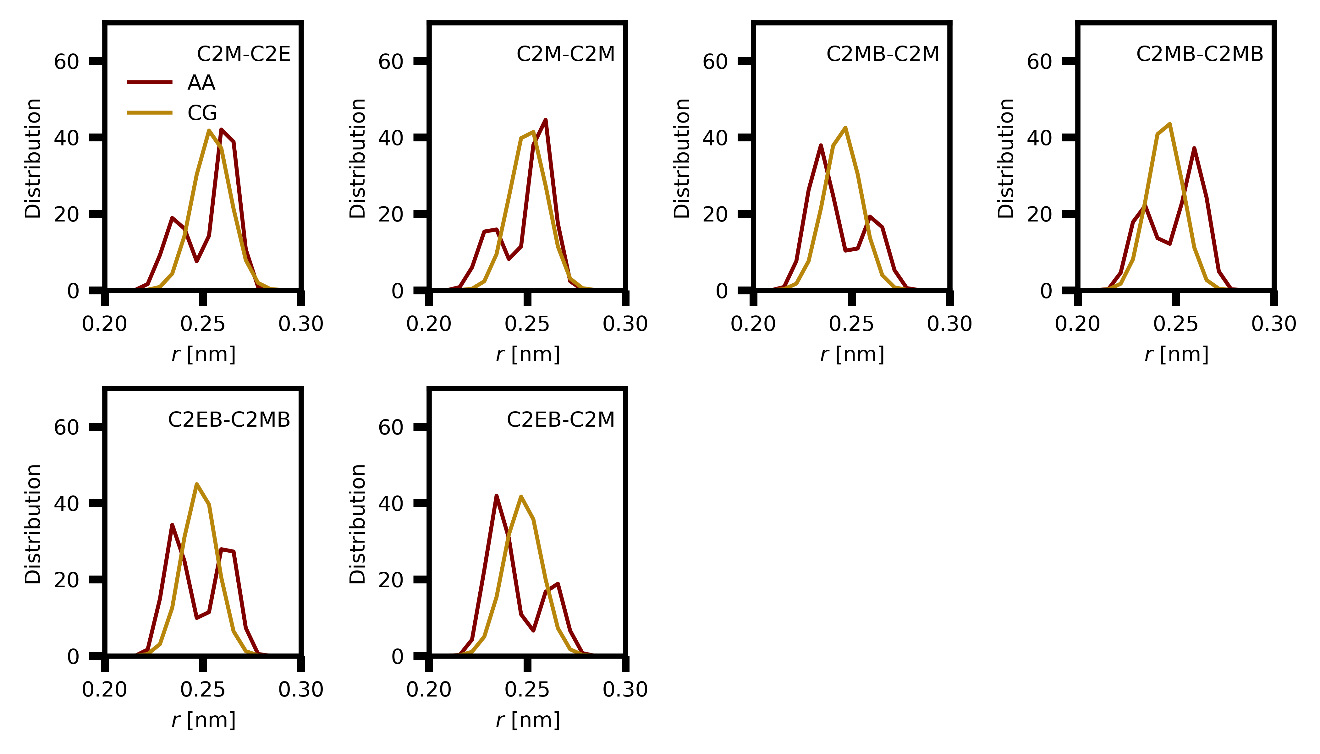


Fig. S3. Comparison of bond-distance distribution between the all-atom model (AA) and coarse-grained model (CG). Center of mass is calculated for the all-atom model to get position of coarse-grained particles.


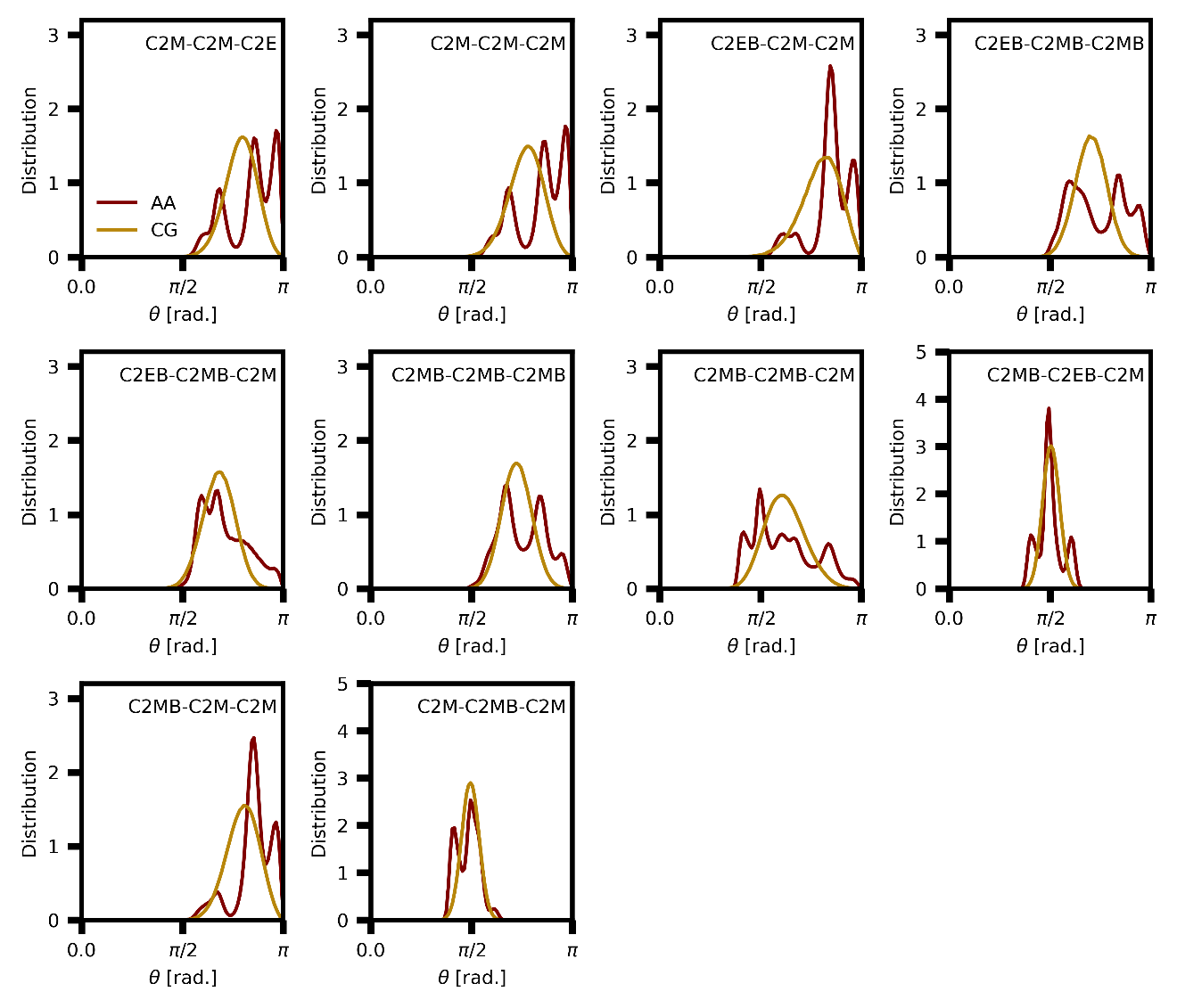


Fig. S4. Comparison of bond angle distribution between the all-atom model (AA) and coarse-grained model (CG). Center of mass is calculated for the all-atom model to get position of coarse-grained particles.


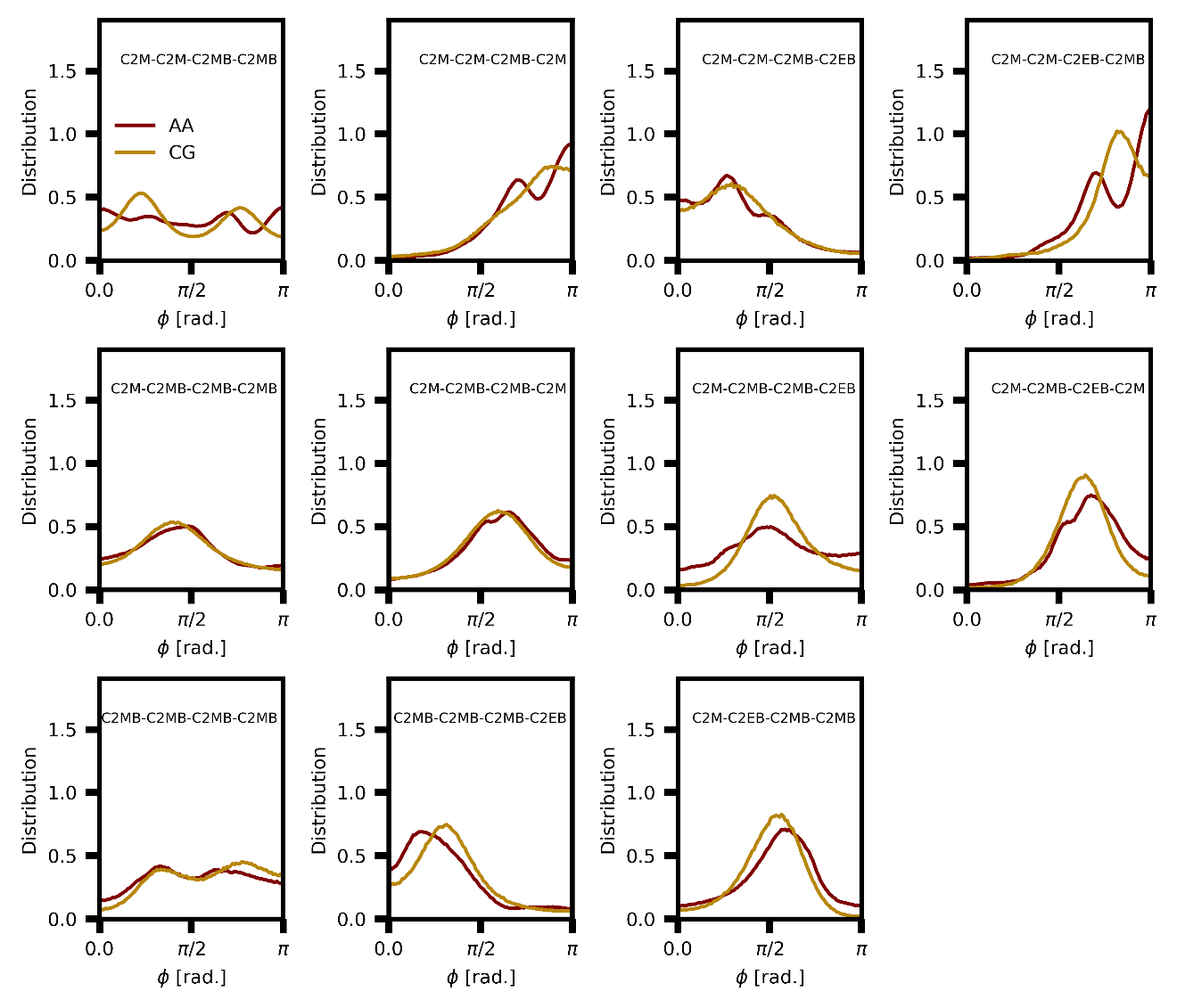


Fig. S5. Comparison of dihedral angle distribution between the all-atom model (AA) and coarse-grained model (CG). Center of mass is calculated for the all-atom model to get position of coarse-grained particles.


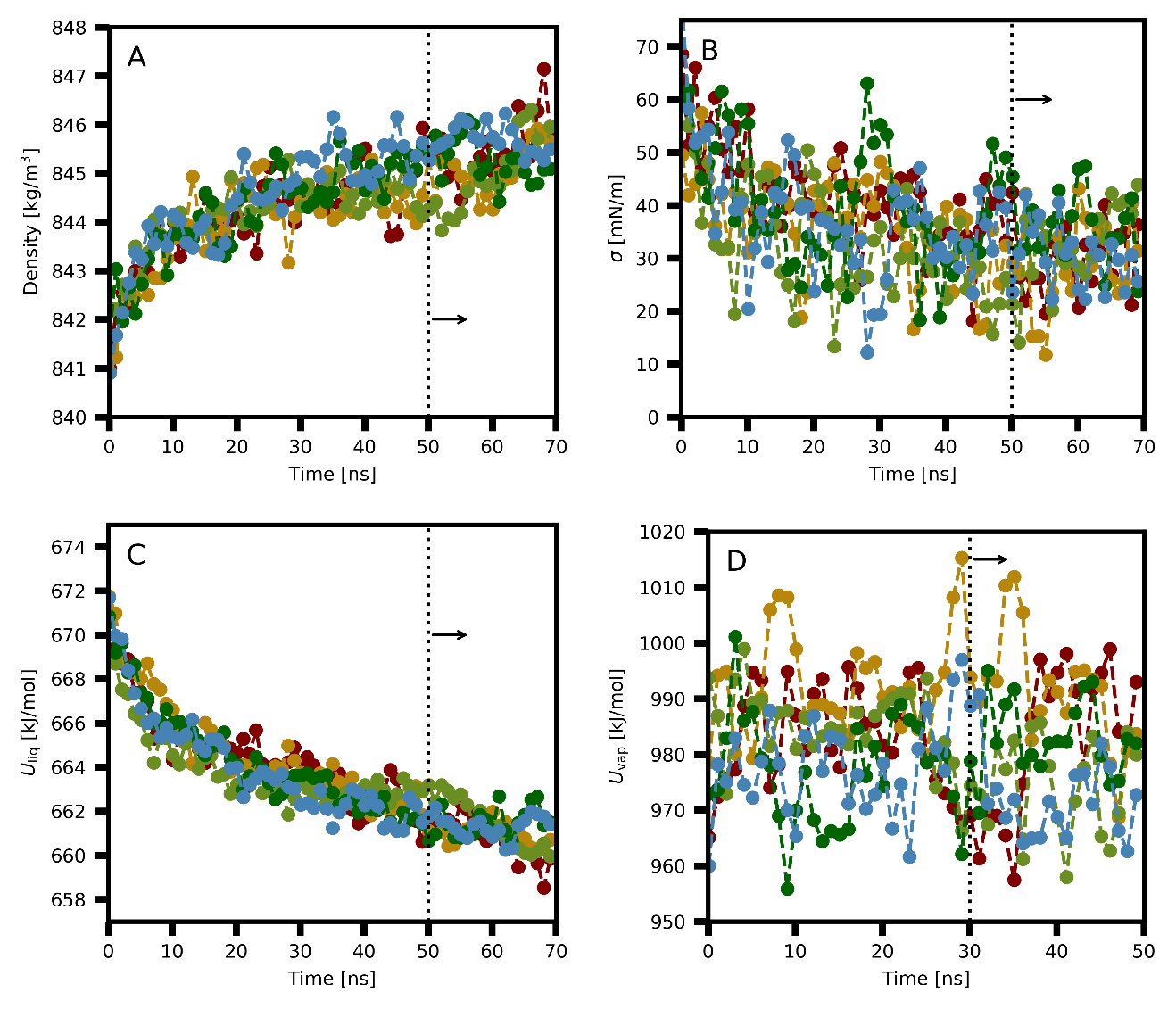


Fig. S6. Time evolutions of (A) density, (B) surface tension, (C) potential energy of liquid phase, and (D) potential energy of vapor phase calculated by five independent all-atom molecular dynamics simulations for poly 1-decene with nine repeating units. Properties in Table S5 are obtained from the average of the last 20 ns calculations represented by dotted lines and arrows.


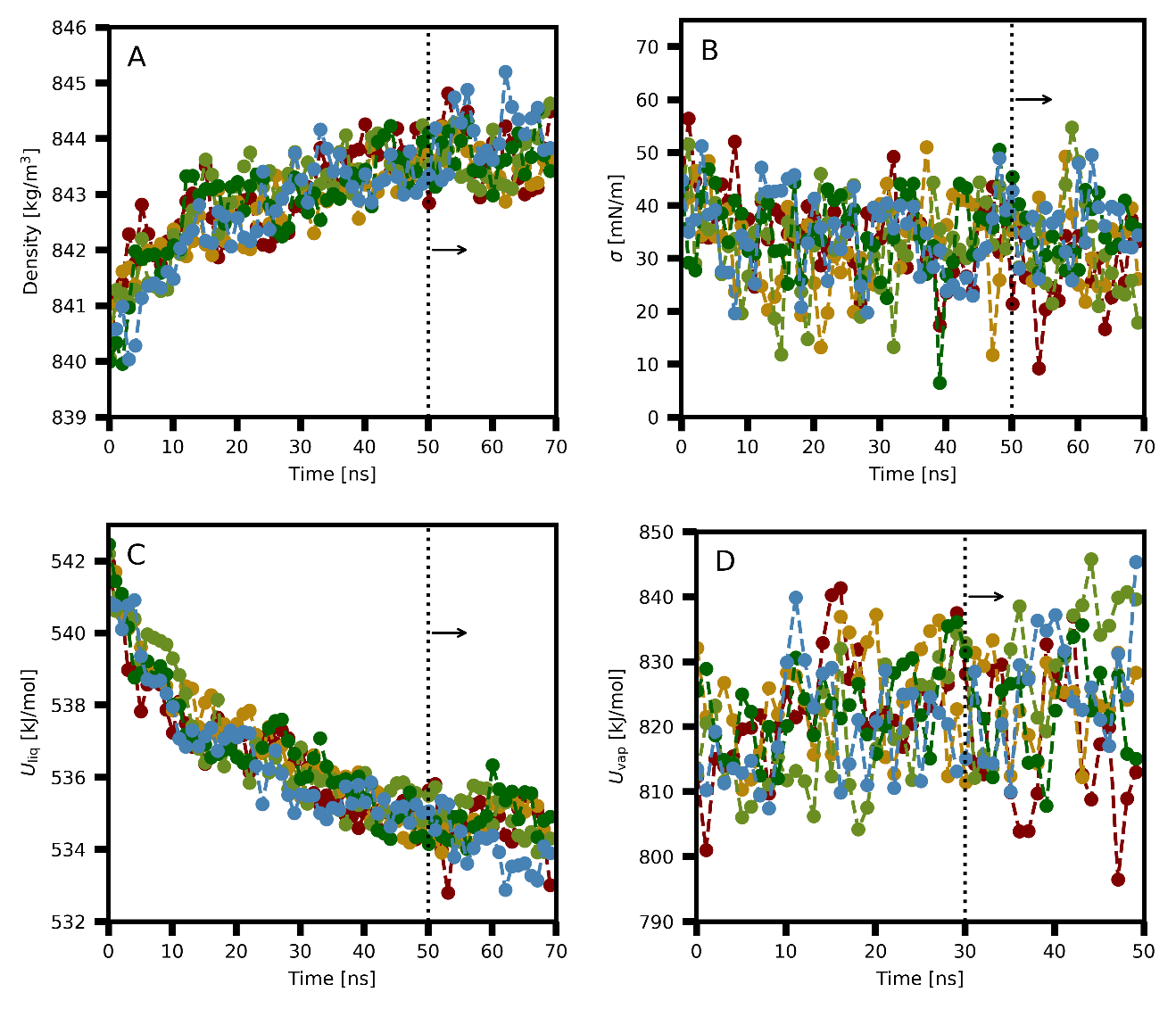


Fig. S7. Time evolutions of (A) density, (B) surface tension, (C) potential energy of liquid phase, and (D) potential energy of vapor phase calculated by five independent all-atom molecular dynamics simulations for poly 1-dodecene with six repeating units. Properties in Table S5 are obtained from the average of the last 20 ns calculations represented by dotted lines and arrows.


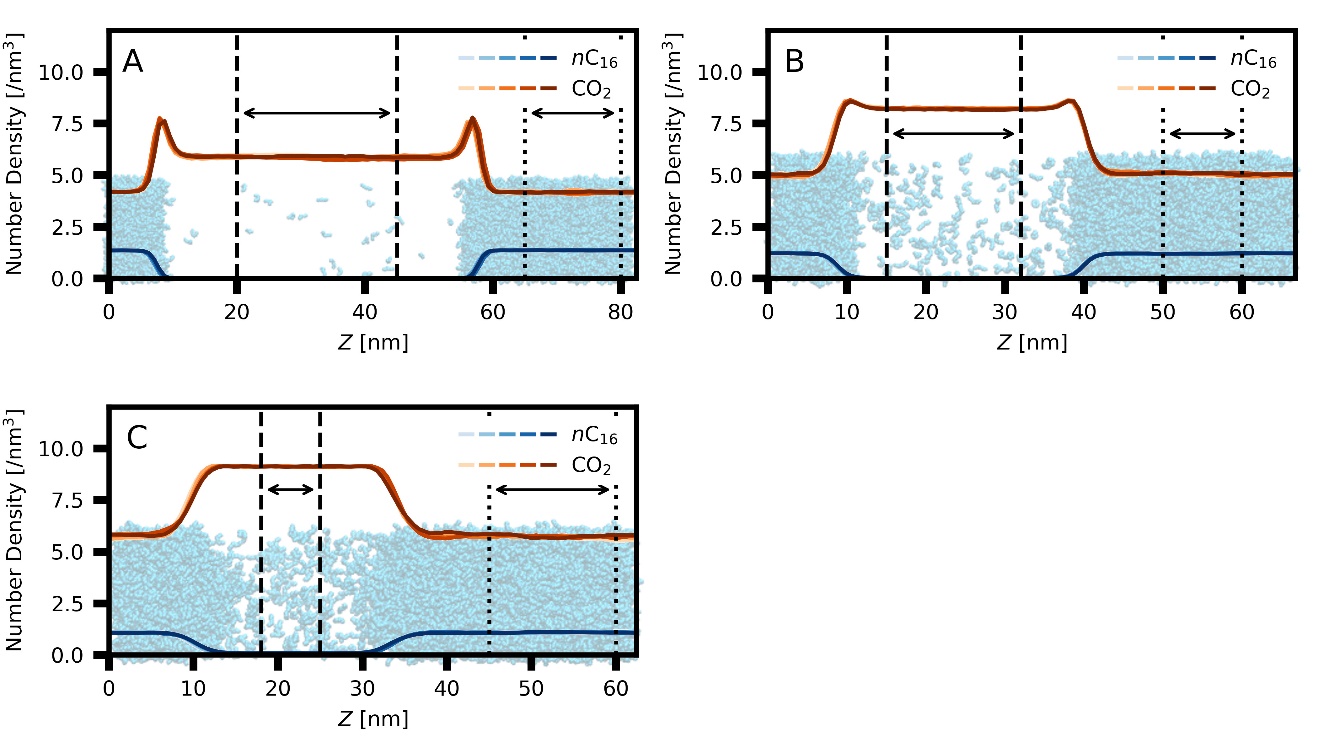


Fig. S8. Number density profiles normal to the interface for the system of *n*-hexadecane (*n*C_16_) and CO_2_ for mixing parameter zeta ($\boldsymbol{\zeta}$) of 0.95, at 344.3 K, and at pressures of (A) 12 MPa, (B) 15 MPa, (C) 18 MPa. Color intensity represents time evolution every 50 ns blocks, namely 50 ns $\boldsymbol{\times}$ 5 blocks. Areas between dashed lines and dotted lines define CO_2_-rich phase, and *n*-hexadecane-rich phase.


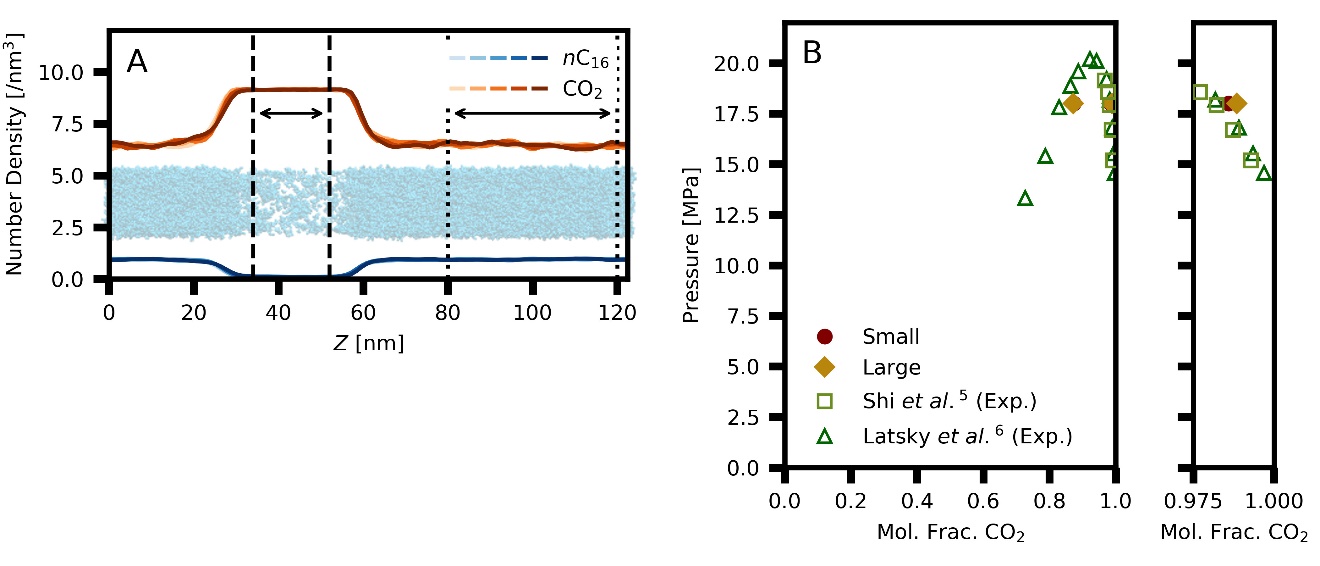


Fig. S9. System size dependency of mutual solubility investigated for the system of *n*-hexadecane and CO_2_ at a pressure of 18 MPa and at 344.3 K. (A) Number density profiles normal to the interface for the system of *n*-hexadecane and CO_2_ for the mixing parameter zeta ($\boldsymbol{\zeta}$) of 0.96. Color intensity represents time evolution every 50 ns blocks, namely 50 ns $\boldsymbol{\times}$ 5 blocks. Areas between dashed lines and dotted lines define CO_2_-rich phase, and *n*-hexadecane-rich phase, respectively. (B) $\boldsymbol{P}$-$\boldsymbol{x}$ diagram of *n*-hexadecane and CO_2_ system for two different system sizes and comparison with experimental data. The right panel represents magnified image of mole fraction of CO_2_ in CO_2_-rich phase (namely, solubility of *n*-hexadecane in CO_2_). The small system consists of 10000 *n*-hexadecane and 100000 CO_2_ molecules. The large system consists of 20000 *n*-hexadecane and 200000 CO_2_ molecules. SEs are smaller than the symbols.


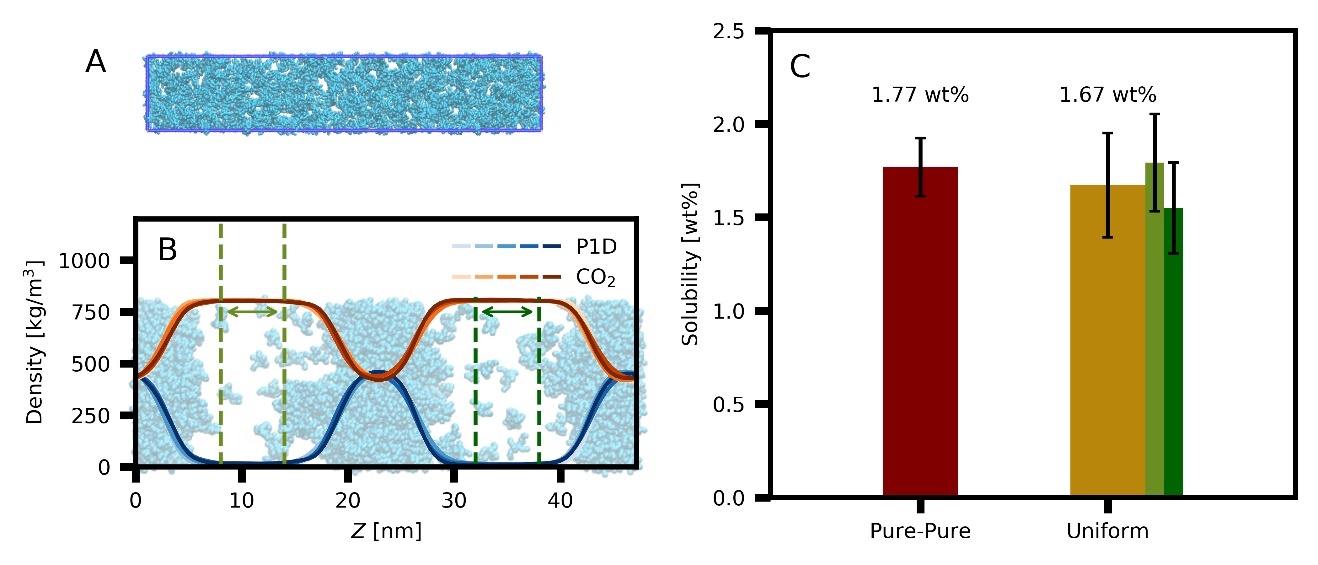


Fig. S10. Initial configuration dependency of solubility evaluated in systems of poly 1-decene with six repeating units at a pressure of 35 MPa and at 344.3 K. (A) Initial configuration of over-saturated uniform configuration, where CO_2_ particles are not shown for clarity. (B) Density profile normal to the interface. Color intensity represents time evolution every 100 ns blocks, namely 100 ns $\boldsymbol{\times}$ 5 blocks. Areas between dashed lines define CO_2_-rich phase from which solubility is calculated. Two CO_2_-rich phases form named as left phase (between dashed light green lines) and right phase (between dashed dark green lines). Snapshots from the last configuration are overlaid, where CO_2_ particles are not shown for clarity. (C) Comparison of solubilities of separate pure-pure initial configuration and over-saturated uniform configuration. Concentrations of the two CO_2_-rich phases are represented by narrow bars. The color corresponds to the color of the dashed lines in (B). The concentrations of the two CO_2_-rich phases are averaged to obtain solubility from over-saturated uniform configuration. Error bars represent SEs.


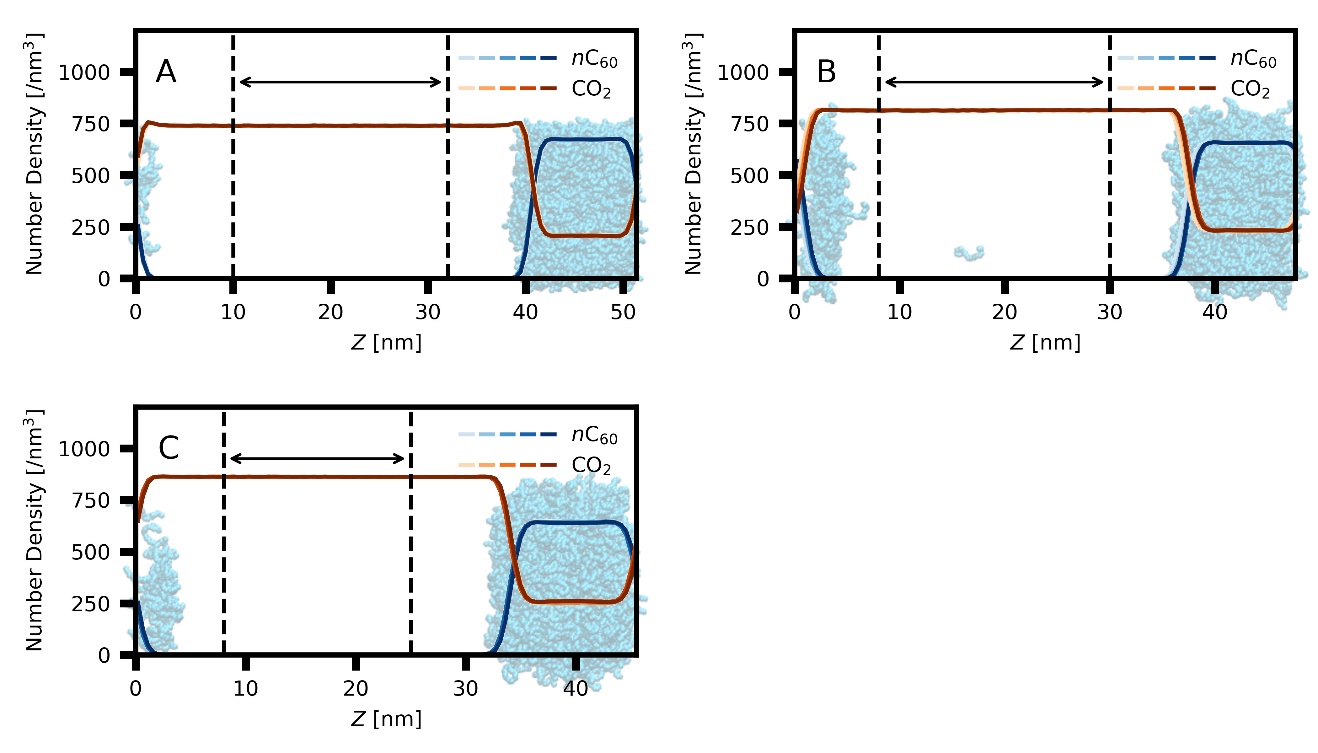


Fig. S11. Density profiles normal to the interface in the system of *n*-hexacontane (*n*C_60_) and CO_2_ at 344.3 K and at pressures of (A) 25 MPa, (B) 35 MPa, (C) 45 MPa. Color intensity represents time evolution every 100 ns blocks, namely 100 ns $\boldsymbol{\times}$ 5 blocks. Areas between dashed lines define CO_2_-rich phase from which solubility is calculated. Snapshots from the last configuration are overlaid; CO_2_ particles are not shown for clarity.


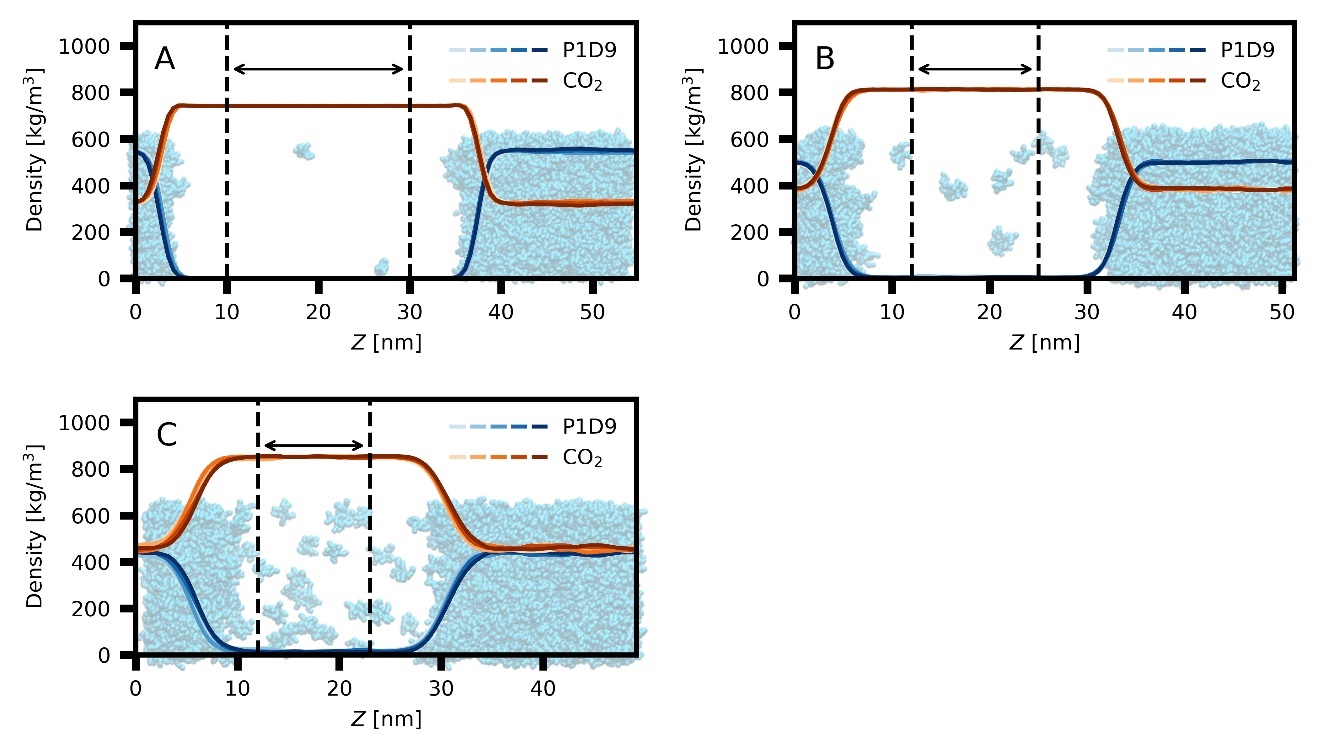


Fig. S12. Density profiles normal to the interface in the system of poly 1-decene with nine repeating units (P1D9) and CO_2_ at 344.3 K and at pressures of (A) 25 MPa, (B) 35 MPa, (C) 45 MPa. Color intensity represents time evolution every 100 ns blocks, namely 100 ns $\boldsymbol{\times}$ 5 blocks. Areas between dashed lines define CO_2_-rich phase from which solubility is calculated. Snapshots from the last configuration are overlaid; CO_2_ particles are not shown for clarity.


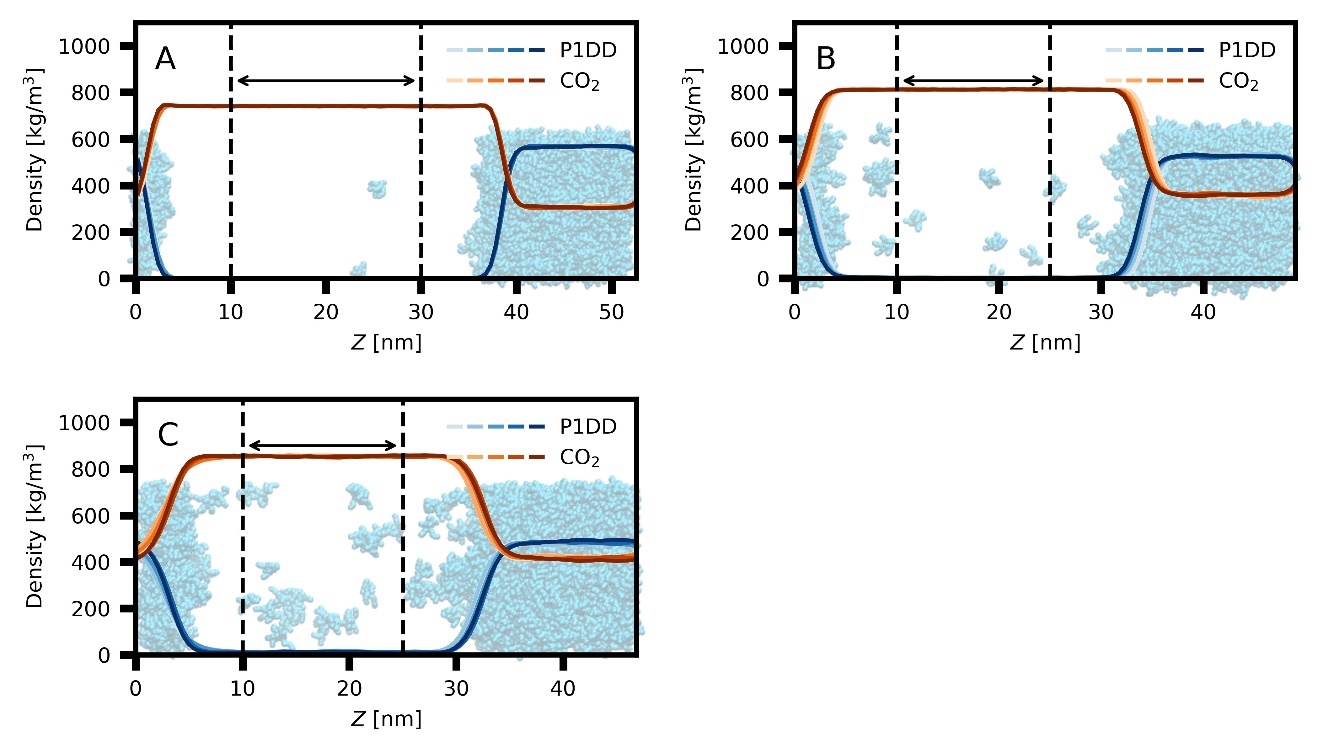


Fig. S13. Density profiles normal to the interface in the system of poly 1-dodecene with six repeating units (P1DD) and CO_2_ at 344.3 K and at pressures of (A) 25 MPa, (B) 35 MPa, (C) 45 MPa. Color intensity represents time evolution every 100 ns blocks, namely 100 ns $\boldsymbol{\times}$ 5 blocks. Areas between dashed lines are defined as CO_2_-rich phase from which solubility is calculated. Snapshots from the last configuration are overlaid; CO_2_ particles are not shown for clarity.


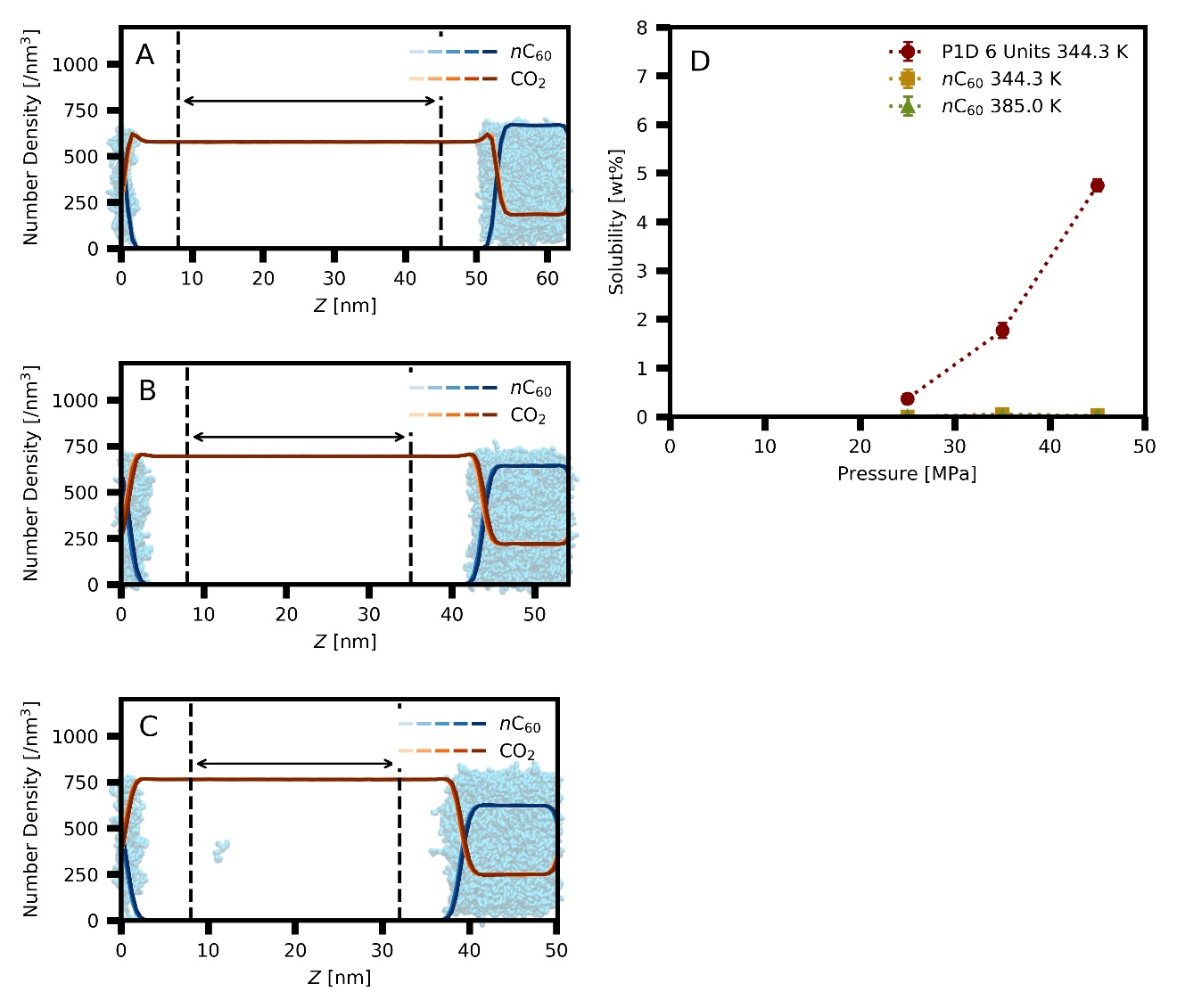


Fig. S14. Temperature dependency of solubility of *n*-hexacontane (*n*C_60_) in CO_2_. (A, B, and C) Density profiles normal to the interface for the system of *n*C_60_ and CO_2_ at 385.0 K, which is above melting point of *n*C_60_, and at pressures of (A) 25 MPa, (B) 35 MPa, (C) 45 MPa. Color intensity represents time evolution every 100 ns blocks, namely 100 ns $\boldsymbol{\times}$ 5 blocks. Areas between dashed lines define CO_2_-rich phase from which solubility is calculated. Snapshots from the last configuration are overlaid; CO_2_ particles are not shown for clarity. (D) Solubility of poly 1-decene with six repeating units at 344.3 K, solubility of *n*C_60_ at 344.3 K and 385.0 K (very close). Solubility of *n*C_60_ at 344.3 K calculated from the density distribution in Fig. S11.


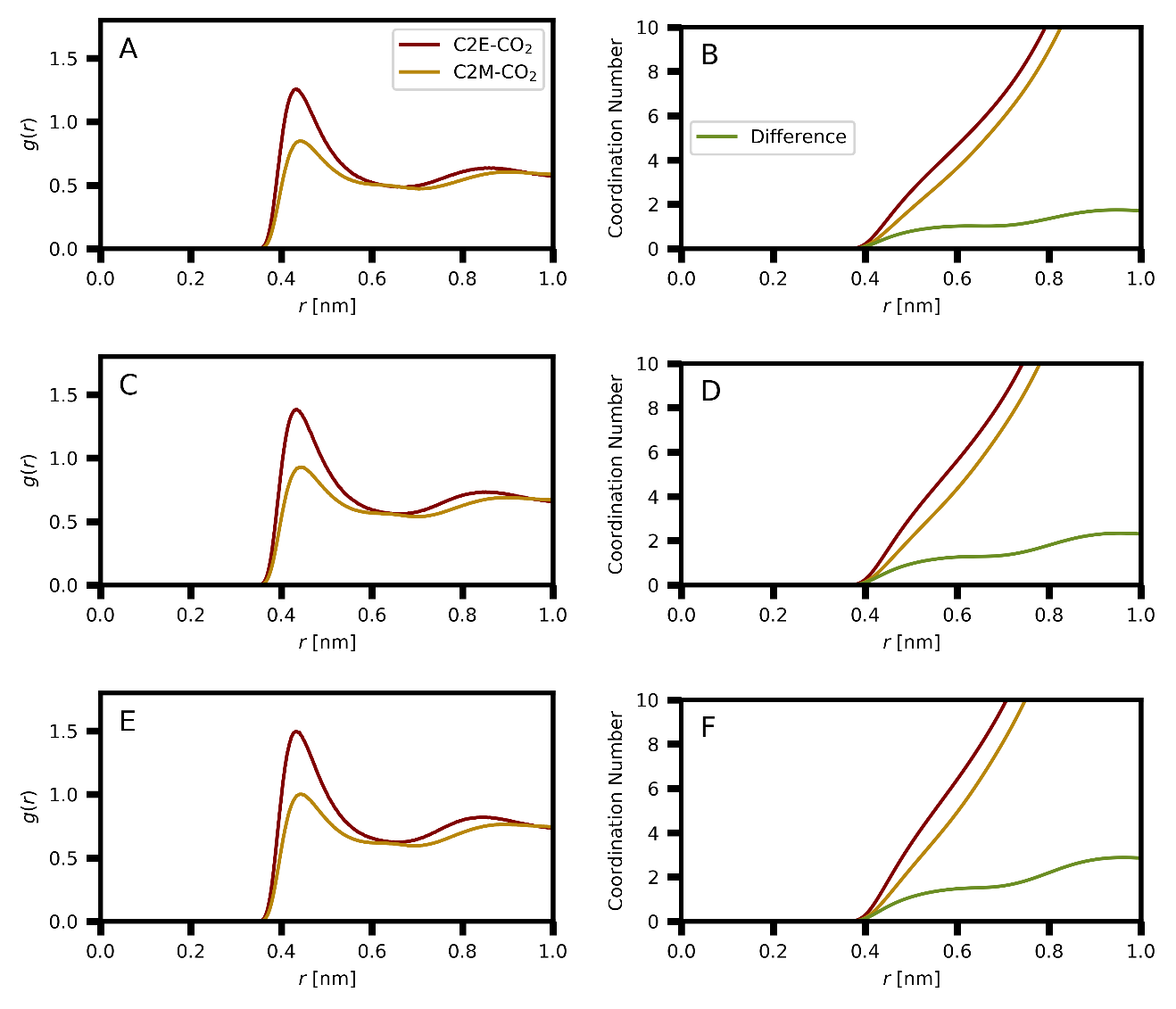


Fig. S15. Radial distribution function ($\boldsymbol{g(r)}$) and coordination numbers for the interfacial systems of poly 1-decene with six repeating units and CO_2_ at 344.3 K: (A, B) 25 MPa, (C, D) 35 MPa, (E, F) 45 MPa. The last 100 ns trajectory is used for the analysis. C2E and C2M represent coarse grained particle types; edge of the chain and middle of the chain in poly 1-decene, respectively, which correspond to the particle types shown in Fig. 1A.


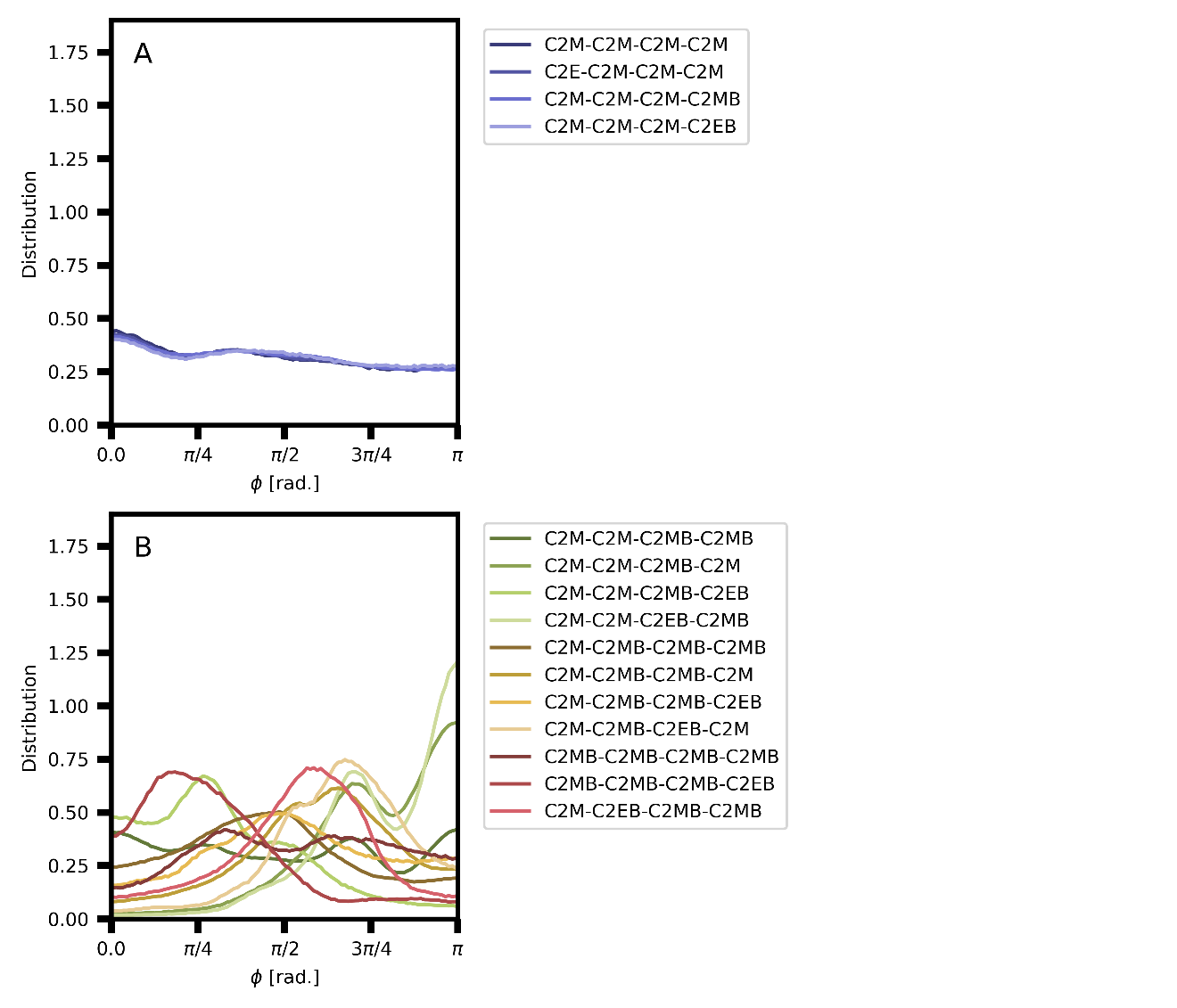


Fig. S16. Dihedral angle distributions from all-atom molecular dynamics simulations. Center of mass is calculated to get position of coarse-grained particles. (A) Dihedral angles showing uniform distributions. The dihedral angle potentials are not imposed for these particles. Dihedral potentials are not considered for these particles in the past study (7). (B) Dihedral distributions showing non-uniformity. Dihedral potentials are imposed for these particles.

Table S1. Coarse-grained non-bonded parameters for poly 1-decene oligomers.

| Particle Type | $\epsilon$ [kJ/mol] | $\sigma$ [nm] |
| --- | --- | --- |
| C2E | 1.552264 | 0.4337429499 |
| C2M | 1.430928 | 0.4337429499 |
| C2EB | 0.7203806 | 0.4915705964 |
| C2MB | 0.6039957 | 0.4565614875 |

Table S2. Coarse-grained bonded parameters for poly 1-decene oligomers.

| Bond Type | $b_{ij}$ [nm] | $k_{ij}^{b}$ [kJ/mol∙nm^2^] |
| --- | --- | --- |
| C2E-C2M | 0.2561 | 29547.65253 |
| C2M-C2M | 0.2520 | 30649.42089 |
| C2EB-C2M | 0.2495 | 28976.49200 |
| C2EB-C2MB | 0.2496 | 34674.70785 |
| C2MB-C2MB | 0.2455 | 33449.53152 |
| C2MB-C2M | 0.2468 | 30597.74712 |

Table S3. Coarse-grained angle parameters for poly 1-decene oligomers.

| Angle Type | $\theta_{ijk}^{0}$ [deg.] | $k_{ijk}^{\theta}$ [kJ/mol∙rad^2^] |
| --- | --- | --- |
| C2E-C2M-C2M | 147.766 | 32.5339806 |
| C2M-C2M-C2M | 144.226 | 28.9674660 |
| C2EB-C2M-C2M | 157.523 | 17.9018253 |
| C2EB-C2MB-C2MB | 137.537 | 27.5043766 |
| C2EB-C2MB-C2M | 135.879 | 30.8005018 |
| C2MB-C2MB-C2MB | 138.400 | 32.9387207 |
| C2MB-C2MB-C2M | 121.622 | 16.9990669 |
| C2MB-C2EB-C2M | 91.282 | 139.9138127 |
| C2MB-C2M-C2M | 151.655 | 27.9325047 |
| C2M-C2MB-C2M | 90.577 | 141.0148637 |

Table S4. Coarse-grained dihedral parameters for poly 1-decene oligomers.

| Dihedral Type | $n$ [-] | $k_{n}^{\phi}$ [kJ/mol] | $\phi_{n}^{s}$ [deg.] |
| --- | --- | --- | --- |
| C2M-C2M-C2MB-C2MB | 1 | 2.8733415 | 90 |
|  | 3 | 1.3460780 | 90 |
|  | 4 | 0.9363449 | 0 |
| C2M-C2M-C2MB-C2M | 1 | 4.3556590 | 0 |
|  | 3 | 1.0016316 | 90 |
|  | 4 | 0.3740166 | 0 |
| C2M-C2M-C2MB-C2EB | 1 | 2.7938509 | 180 |
|  | 2 | 1.0295140 | 0 |
|  | 3 | 0.3038307 | 60 |
|  | 4 | 0.3262739 | 0 |
| C2M-C2M-C2EB-C2MB | 1 | 5.1213635 | 0 |
|  | 3 | 1.8253896 | 90 |
|  | 4 | 0.8124859 | 0 |
| C2M-C2MB-C2MB-C2MB | 1 | 0.6863157 | 180 |
|  | 2 | 0.7690066 | 0 |
|  | 3 | 0.4130208 | 0 |
| C2M-C2MB-C2MB-C2M | 1 | 0.1584367 | 0 |
|  | 2 | 1.5392127 | 0 |
|  | 3 | 0.3978295 | 180 |
|  | 4 | 0.0841112 | 0 |
| C2M-C2MB-C2MB-C2EB | 1 | 0.3725118 | 0 |
|  | 2 | 1.0206721 | 0 |
|  | 3 | 0.2960292 | 0 |
|  | 4 | 0.1476544 | 180 |
| C2M-C2MB-C2EB-C2M | 1 | 3.0538864 | 0 |
|  | 2 | 1.6787437 | 0 |
|  | 3 | 0.8509530 | 180 |
|  | 4 | 0.0962368 | 0 |
| C2MB-C2MB-C2MB-C2MB | 1 | 0.4791121 | 0 |
|  | 2 | 0.4406693 | 0 |
|  | 3 | 0.4290069 | 0 |
|  | 4 | 0.5718495 | 0 |
| C2MB-C2MB-C2MB-C2EB | 1 | 2.8698920 | 180 |
|  | 2 | 0.1594448 | 0 |
|  | 3 | 0.8121177 | 0 |
|  | 4 | 0.1394220 | 0 |
| C2M-C2EB-C2MB-C2MB | 1 | 0.5981151 | 0 |
|  | 2 | 2.0068896 | 0 |
|  | 3 | 0.7781588 | 160 |
|  | 4 | 0.2219327 | 0 |

Table S5. Properties of poly 1-decene with nine repeating units (P1D9) and poly 1-dodecene with six repeating units (P1DD) from the all-atom model (CGenFF) (8-10) and the coarse-grained model in this study. Error represents SEs.

| Compounds | Properties | All-atom model | Coarse-grained model | Diff. [%] |
| --- | --- | --- | --- | --- |
| P1D9 | Density [kg/m^3^] | 845.3$\pm$0.3 | 846.24$\pm$0.01 | 0.1 |
|  | Surface Tension [mN/m] | 31$\pm$2 | 31.1$\pm$0.3 | 0.3 |
|  | Enthalpy of Vaporization [kJ/mol] | 322$\pm$7 | 330.8$\pm$0.3 | 2.7 |
| P1DD | Density [kg/m^3^] | 843.7$\pm$0.2 | 846.37$\pm$0.03 | 0.3 |
|  | Surface Tension [mN/m] | 33$\pm$2 | 32.6$\pm$0.6 | 1.2 |
|  | Enthalpy of Vaporization [kJ/mol] | 291$\pm$4 | 292.9$\pm$0.2 | 0.7 |

Table S6. Initial guess and boundary of coarse-grained parameters.

| Parameter | Initial Guess | Min. | Max. |
| --- | --- | --- | --- |
| $\epsilon$ [C2EB] | Random | 0.2 | 1.5 |
| $\sigma$ [C2EB] | Random | 0.35 | 0.7 |
| $\epsilon$ [C2MB] | Random | 0.2 | 1.5 |
| $\sigma$ [C2MB] | Random | 0.35 | 0.7 |
| $b_{ij}$ [C2EB-C2M] | 0.24861953 | $\times0.9$ | $\times1.1$ |
| $k_{ij}^{b}$[C2EB-C2M] | 2.88878852e+4 | $\times0.9$ | $\times1.1$ |
| $b_{ij}$ [C2EB-C2MB] | 0.249470034 | $\times0.9$ | $\times1.1$ |
| $k_{ij}^{b}$[C2EB-C2MB] | 3.47880574e+4 | $\times0.9$ | $\times1.1$ |
| $b_{ij}$ [C2MB-C2MB] | 0.246433357 | $\times0.9$ | $\times1.1$ |
| $k_{ij}^{b}$[C2MB-C2MB] | 3.33241761e+4 | $\times0.9$ | $\times1.1$ |
| $b_{ij}$ [C2MB-C2M] | 0.246655605 | $\times0.9$ | $\times1.1$ |
| $k_{ij}^{b}$[C2MB-C2M] | 3.05460603e+4 | $\times0.9$ | $\times1.1$ |
| $\theta_{ijk}^{0}$ [C2EB-C2M-C2M] | 157.3318087 | $\times0.9$ | $\times1.1$ |
| $k_{ijk}^{\theta}$ [C2EB-C2M-C2M] | 17.94580776 | $\times0.9$ | $\times1.1$ |
| $\theta_{ijk}^{0}$ [C2EB-C2MB-C2MB] | 138.0039186 | $\times0.9$ | $\times1.1$ |
| $k_{ijk}^{\theta}$ [C2EB-C2MB-C2MB] | 27.40202433 | $\times0.9$ | $\times1.1$ |
| $\theta_{ijk}^{0}$ [C2EB-C2MB-C2M] | 135.4359303 | $\times0.9$ | $\times1.1$ |
| $k_{ijk}^{\theta}$ [C2EB-C2MB-C2M] | 30.775913 | $\times0.9$ | $\times1.1$ |
| $\theta_{ijk}^{0}$ [C2MB-C2MB-C2MB] | 138.6393185 | $\times0.9$ | $\times1.1$ |
| $k_{ijk}^{\theta}$ [C2MB-C2MB-C2MB] | 32.95807282 | $\times0.9$ | $\times1.1$ |
| $\theta_{ijk}^{0}$ [C2MB-C2MB-C2M] | 121.2303259 | $\times0.9$ | $\times1.1$ |
| $k_{ijk}^{\theta}$ [C2MB-C2MB-C2M] | 16.97173048 | $\times0.9$ | $\times1.1$ |
| $\theta_{ijk}^{0}$ [C2MB-C2EB-C2M] | 91.20995928 | $\times0.9$ | $\times1.1$ |
| $k_{ijk}^{\theta}$ [C2MB-C2EB-C2M] | 139.60001509 | $\times0.9$ | $\times1.1$ |
| $\theta_{ijk}^{0}$ [C2MB-C2M-C2M] | 151.3927893 | $\times0.9$ | $\times1.1$ |
| $k_{ijk}^{\theta}$ [C2MB-C2M-C2M] | 27.99635512 | $\times0.9$ | $\times1.1$ |
| $\theta_{ijk}^{0}$ [C2M-C2MB-C2M] | 90.19622849 | $\times0.9$ | $\times1.1$ |
| $k_{ijk}^{\theta}$ [C2M-C2MB-C2M] | 140.97454785 | $\times0.9$ | $\times1.1$ |
| $\phi_{1}^{s}$ [C2M-C2M-C2MB-C2MB] | 2.86357997 | $\times0.9$ | $\times1.1$ |
| $\phi_{3}^{s}$ [C2M-C2M-C2MB-C2MB] | 1.34381414 | $\times0.9$ | $\times1.1$ |
| $\phi_{4}^{s}$ [C2M-C2M-C2MB-C2MB] | 9.33411647e-1 | $\times0.9$ | $\times1.1$ |
| $\phi_{1}^{s}$ [C2M-C2M-C2MB-C2M] | 4.355788 | $\times0.9$ | $\times1.1$ |
| $\phi_{3}^{s}$ [C2M-C2M-C2MB-C2M] | 1.0010453 | $\times0.9$ | $\times1.1$ |
| $\phi_{4}^{s}$ [C2M-C2M-C2MB-C2M] | 3.75743553e-1 | $\times0.9$ | $\times1.1$ |
| $\phi_{1}^{s}$ [C2M-C2M-C2MB-C2EB] | 2.78547895 | $\times0.9$ | $\times1.1$ |
| $\phi_{2}^{s}$ [C2M-C2M-C2MB-C2EB] | 1.02912694 | $\times0.9$ | $\times1.1$ |
| $\phi_{3}^{s}$ [C2M-C2M-C2MB-C2EB] | 3.03564358e-1 | $\times0.9$ | $\times1.1$ |
| $\phi_{4}^{s}$ [C2M-C2M-C2MB-C2EB] | 3.25715622e-1 | $\times0.9$ | $\times1.1$ |
| $\phi_{1}^{s}$ [C2M-C2M-C2EB-C2MB] | 5.12988704 | $\times0.9$ | $\times1.1$ |
| $\phi_{3}^{s}$ [C2M-C2M-C2EB-C2MB] | 1.82140666 | $\times0.9$ | $\times1.1$ |
| $\phi_{4}^{s}$ [C2M-C2M-C2EB-C2MB] | 8.12466786e-1 | $\times0.9$ | $\times1.1$ |
| $\phi_{1}^{s}$ [C2M-C2MB-C2MB-C2MB] | 6.86207819e-1 | $\times0.9$ | $\times1.1$ |
| $\phi_{2}^{s}$ [C2M-C2MB-C2MB-C2MB] | 7.7279522e-1 | $\times0.9$ | $\times1.1$ |
| $\phi_{3}^{s}$ [C2M-C2MB-C2MB-C2MB] | 4.13015717e-1 | $\times0.9$ | $\times1.1$ |
| $\phi_{1}^{s}$ [C2M-C2MB-C2MB-C2M] | 1.59063844e-1 | $\times0.9$ | $\times1.1$ |
| $\phi_{2}^{s}$ [C2M-C2MB-C2MB-C2M] | 1.53592923 | $\times0.9$ | $\times1.1$ |
| $\phi_{3}^{s}$ [C2M-C2MB-C2MB-C2M] | 3.95404325e-1 | $\times0.9$ | $\times1.1$ |
| $\phi_{4}^{s}$ [C2M-C2MB-C2MB-C2M] | 8.38342061e-2 | $\times0.9$ | $\times1.1$ |
| $\phi_{1}^{s}$ [C2M-C2MB-C2MB-C2EB] | 3.73212022e-1 | $\times0.9$ | $\times1.1$ |
| $\phi_{2}^{s}$ [C2M-C2MB-C2MB-C2EB] | 1.02327917 | $\times0.9$ | $\times1.1$ |
| $\phi_{3}^{s}$ [C2M-C2MB-C2MB-C2EB] | 2.95233122e-1 | $\times0.9$ | $\times1.1$ |
| $\phi_{4}^{s}$ [C2M-C2MB-C2MB-C2EB] | 1.47691303e-1 | $\times0.9$ | $\times1.1$ |
| $\phi_{1}^{s}$ [C2M-C2MB-C2EB-C2M] | 3.06152446 | $\times0.9$ | $\times1.1$ |
| $\phi_{2}^{s}$ [C2M-C2MB-C2EB-C2M] | 1.67550146 | $\times0.9$ | $\times1.1$ |
| $\phi_{3}^{s}$ [C2M-C2MB-C2EB-C2M] | 8.52877735e-1 | $\times0.9$ | $\times1.1$ |
| $\phi_{4}^{s}$ [C2M-C2MB-C2EB-C2M] | 9.62622908e-2 | $\times0.9$ | $\times1.1$ |
| $\phi_{1}^{s}$ [C2MB-C2MB-C2MB-C2MB] | 4.81233344e-1 | $\times0.9$ | $\times1.1$ |
| $\phi_{2}^{s}$ [C2MB-C2MB-C2MB-C2MB] | 4.40060484e-1 | $\times0.9$ | $\times1.1$ |
| $\phi_{3}^{s}$ [C2MB-C2MB-C2MB-C2MB] | 4.29242159e-1 | $\times0.9$ | $\times1.1$ |
| $\phi_{4}^{s}$ [C2MB-C2MB-C2MB-C2MB] | 5.72508771e-1 | $\times0.9$ | $\times1.1$ |
| $\phi_{1}^{s}$ [C2MB-C2MB-C2MB-C2EB] | 2.86527876 | $\times0.9$ | $\times1.1$ |
| $\phi_{2}^{s}$ [C2MB-C2MB-C2MB-C2EB] | 1.5996107e-1 | $\times0.9$ | $\times1.1$ |
| $\phi_{3}^{s}$ [C2MB-C2MB-C2MB-C2EB] | 8.11854945e-1 | $\times0.9$ | $\times1.1$ |
| $\phi_{4}^{s}$ [C2MB-C2MB-C2MB-C2EB] | 1.39558314e-1 | $\times0.9$ | $\times1.1$ |
| $\phi_{1}^{s}$ [C2M-C2EB-C2MB-C2MB] | 5.98748315e-1 | $\times0.9$ | $\times1.1$ |
| $\phi_{2}^{s}$ [C2M-C2EB-C2MB-C2MB] | 1.99890395 | $\times0.9$ | $\times1.1$ |
| $\phi_{3}^{s}$ [C2M-C2EB-C2MB-C2MB] | 7.75416747e-1 | $\times0.9$ | $\times1.1$ |
| $\phi_{4}^{s}$ [C2M-C2EB-C2MB-C2MB] | 2.21871343e-1 | $\times0.9$ | $\times1.1$ |

**SI References**

1. G. J. Besserer, D. B. Robinson, Equilibrium-phase properties of *n*-pentane-carbon dioxide system. *J. Chem. Eng. Data* 18, 416–419 (1973).
2. H. Cheng, M. E. P. Fernández, J. A. Zollweg, W. B. Streett, Vapor-liquid equilibrium in the system carbon dioxide + *n*-pentane from 252 to 458 K at pressures to 10 MPa. *J. Chem. Eng. Data* 34, 319–323 (1989).
3. G. J. Besserer, D. B. Robinson, Equilibrium-phase properties of isopentane-carbon dioxide system. *J. Chem. Eng. Data* 20, 93–96 (1975).
4. N. N. Shah, M. E. P. Fernández, J. A. Zollweg, W. B. Streett, Vapor-liquid equilibrium in the system carbon dioxide + 2,2-dimethylpropane from 262 to 424 K at pressures to 8.4 MPa. *J. Chem. Eng. Data* 35, 278–283 (1990).
5. Q. Shi, L. Jing, W. Qiao, Solubility of *n*-alkanes in supercritical CO_2_ at diverse temperature and Pressure. *J.* CO_2_ *Util.* 9, 29–38 (2015).
6. C. Latsky, B. Cordeiro, C. E. Schwartz, High pressure bubble- and dew-point data for systems containing CO_2_ with 1-decanol and *n*-hexadecane. *Fluid Phase Equilib.* 521, 112702 (2020).
7. Y. An, K. K. Bejagam, S. A. Deshmukh, Development of new transferable coarse-grained models of hydrocarbons. *J. Phys. Chem. B* 122, 7143–7153 (2018).
8. K. Vanommeslaeghe, E. Hatcher, C. Acharya, S. Kundu, S. Zhong, J. Shim, E. Darian, O. Guvench, P. Lopes, I. Vorobyov, A. D. MacKerell Jr., CHARMM General Force Field: A Force field for Drug-Like Molecules Compatible with the CHARMM All-Atom Additive Biological Force Field. *J. Comput. Chem.* 31, 671–690 (2010).
9. K. Vanommeslaeghe, A. D. MacKerell Jr., Automation of the CHARMM General Force Field (CGenFF) I: bond perception and atom typing. *J. Chem. Inf. Model.* 52, 3144–3154 (2012).
10. K. Vanommeslaeghe, E. P. Raman, A. D. MacKerell Jr., Automation of the CHARMM General Force Field (CGenFF) II: Assignment of bonded parameters and partial atomic charges. *J. Chem. Inf. Model.* 52, 3155–3168 (2012).
